# Mechanical Learning for Prediction of Sepsis-Associated Encephalopathy

**DOI:** 10.3389/fncom.2021.739265

**Published:** 2021-11-16

**Authors:** Lina Zhao, Yunying Wang, Zengzheng Ge, Huadong Zhu, Yi Li

**Affiliations:** ^1^State Key Laboratory of Complex Severe and Rare Diseases, Emergency Department, Peking Union Medical College Hospital, Peking Union Medical College, Chinese Academy of Medical Sciences, Beijing, China; ^2^Department of Critical Care Medicine, Chifeng Municipal Hospital, Inner Mongolia, China

**Keywords:** sepsis, sepsis-associated encephalopathy, encephalopathy, delirium, nomogram, mechanical learning

## Abstract

**Objective:** The study aims to develop a mechanical learning model as a predictive model for predicting the appearance of sepsis-associated encephalopathy (SAE).

**Materials and Methods:** The prediction model was developed in a primary cohort of 2,028 sepsis patients from June 2001 to October 2012, retrieved from the Medical Information Mart for Intensive Care (MIMIC III) database. Least absolute shrinkage and selection operator (LASSO) regression model was used for data dimension reduction and feature selection. The model was developed using multivariable logistic regression analysis. The performance of the nomogram has been evaluated in terms of calibration, discrimination, and clinical utility.

**Results:** There were nine particular features in septic patients that were significantly associated with SAE. Predictors of individualized prediction nomograms included age, rapid sequential evaluation of organ failure (qSOFA), and drugs including carbapenem antibiotics, quinolone antibiotics, steroids, midazolam, H_2_-antagonist, diphenhydramine hydrochloride, and heparin sodium injection. The area under the curve (AUC) was 0.743, indicating good discrimination. The prediction model showed calibration curves with minor deviations from the ideal predictions. Decision curve analysis (DCA) suggested that the nomogram was clinically useful.

**Conclusion:** We propose a nomogram for the individualized prediction of SAE with satisfactory performance and clinical utility, which could aid the clinician in the early detection and management of SAE.

## Introduction

Sepsis-associated encephalopathy (SAE) is defined as diffuse brain dysfunction caused by a dysregulated host response without central nervous system (CNS) infection (Gofton and Young, 2012). Symptoms and signs range from mild inattentiveness or disorientation, agitation, and hypersomnolence to more severe disturbance of consciousness and coma (Chung et al., 2020). Approximately 70% of the patients with bacteremia display neurological symptoms or signs ranging from lethargy to coma (Peidaee et al., 2018). SAE is associated with increased mortality, prolonged hospitalizations, and inpatient costs. It is also associated with higher severity on the Glasgow Coma Scale (GCS), sequential organ failure assessment score (SOFA), quick sequential organ failure assessment (qSOFA), the simplified acute physiology score (APACHE II) of patients followed by persistent cognitive and functional impairments (Iwashyna et al., 2010; Sonneville et al., 2017). Iwashyna et al. (2010) found that up to 70% of sepsis survivors exhibited lasting neurological impairment, including alterations in mood, cognition, and motor function, and up to 45% had neurocognitive impairments 1 year later. With a mortality rate of up to 63% (Eidelman et al., 1996), and morbidities mentioned above, SAE can have a major effect on the healthcare system, the economy, and the society.

Sepsis-associated encephalopathy is a diagnosis of exclusion. It is diagnosed in the absence of direct infection of the central nervous system, multi-organ failure, traumatic brain injury, fat embolism, and ingestion of illicit drugs (Iacobone et al., 2009). Due to sepsis complications and the lack of an early diagnosis system, diagnosis and management of SAE are often delayed, leading to significant morbidity and mortality. Early diagnosis and treatment for brain injury are crucial for the survival and prognosis of sepsis patients. Sonneville et al. (2017) reported that acute renal failure, hypoglycemia (< 3 mmol/L), hyperglycemia (> 10 mmol/L), hypercapnia (> 45 mmHg), hypernatremia (> 145 mmol/L), and *Staphylococcus aureus* infection were associated with the development of SAE. Recently, Yang et al. (2020) developed a nomogram to forecast mortality in patients with known SAE. However, to the best of our knowledge, there is currently no prediction model for the diagnosis of SAE. This study is the first attempt to establish a predictive nomogram for SAE, based on the sociodemographic and clinical data of 2,535 sepsis patients, to allow for individualized screening for SAE among septic patients.

The study aims to identify early and potential risk factors for SAE by a retrospective analysis of a large clinical database, and establish a comprehensive prediction model for SAE patients. The proposed nomogram can assist in clinical decision-making and identify sepsis patients at high risk for SAE, who should undergo further investigative tests, thus promoting early diagnosis and management of SAE.

## Materials and Methods

### Data Source

Medical Information Mart for Intensive Care (MIMIC III) between June 2001 and October 2012 was employed for this study. MIMIC III was approved by the Institutional Review Boards of Beth Israel Deaconess Medical Center and Massachusetts Institute of Technology. There was no requirement for individual patient consent because anonymized health information was used. This is a publicly accessible single-center critical care database containing longitudinal data on 46,520 patients admitted to the ICU. The raw data were extracted using a structure query language (SQL) with Navicat, and further processed with R software.

### Patient Population

Sepsis patients were defined as an infected on discharge according to ICD-9 codes, who were diagnosed as “sepsis”, “severe sepsis”, and “septic shock” and patient’s blood culture were positive. Based on the definition of sepsis 3.0, we included patients with SOFA score ≥ 2. Inclusion criteria were as follows: (1) more than18 years old; (2) admission time > 24 h in the ICU. Exclusion criteria: (1) with primary brain injury (traumatic brain injury, intracerebral hemorrhage, cerebral embolism, ischemic stroke, epilepsy, or intracranial infection and another cerebrovascular disease) (Supplementary Materials 1–5); (2) mental disorders and neurological disease (Supplementary Material 6); (3) chronic alcohol or drug abuse (Supplementary Material 7); (4) metabolic encephalopathy, hepatic encephalopathy, hypertensive encephalopathy, hypoglycemic coma, and other liver disease or kidney disease affecting consciousness (Supplementary Material 8); (5) severe electrolyte imbalances or glycemic disturbances, including hyponatremia (< 120 mmol/l), hyperglycemia (> 180 mg/dl), or hypoglycemia (< 54 mg/dl); (8) partial pressure of CO_2_ (PaCO_2_) ≥ 80 mmHg; (9) patients whose tracheas have been intubated at the time of admission, given analgesic, and sedated; (10) without an evaluation of GCS.

### Sepsis-Associated Encephalopathy

We defined SAE in the study as sepsis with a GCS < 15 on the first day of ICU admission, delirium, cognitive impairment, altered mental status according to the ICD-9 code, and medicating with haloperidol. Altered consciousness caused by other reasons was excluded. GCS has been established as a clinically effective tool for characterizing SAE and distinguishing it from sepsis (Iwashyna et al., 2010). For sedated, postoperative patients or tracheal intubation, ventilator-assisted breathing, theirGCS score were extracted before they were sedated.

### Data Extraction and Management

R statistical software (R foundation for statistical computing, Vienna, Austria) was used to retrieve patient information from the MIMIC III database. The following basic patient data were collected from each patient, including age, sex, admission type, marital status, and mean value of vital signs during the first 24 h of ICU stay, including heart rate, systolic blood pressure, diastolic blood pressure, respiratory rate, and temperature. Since ICU admission, the first laboratory data include alanine aminotransferase, aspartate aminotransferase, partial thromboplastin time, white blood cell count, lymphocyte, neutrophil, monocytes, eosinophils, hemoglobin, platelet, blood urea nitrogen, creatinine, and glucose. SAPS II, qSOFA score, SOFA score assessment of the severity of illness and organ failure on the first day of ICU admission, and GCS were also recorded. Comorbidity as coded and defined in the ICD-9 code (Supplementary Material 9); site of infection (Supplementary Material 10); organ failure (Supplementary Material 11) and ICU stay time, hospital mortality were collected in the study.

### Statistical Analysis

Data distribution was analyzed using the Shapiro–Wilk test. Continuous variables were expressed as the mean ± standard deviation (SD) or the median (interquartile range, IQR); categorical variables were expressed as frequency and percentage. A non-parametric test (Mann–Whitney *U* test or Kruskal–Wallis test) was used for data with non-normal distribution or heterogeneity of variances. Categorical data were compared using the Pearson Chi-squared test.

Least absolute shrinkage and selection operator (LASSO) regression model was used for data dimension reduction and feature selection (Training set). A nomogram was constructed according to the multivariate logistic regression analysis results (Training set), and it was internally validated using a 1,000 bootstrap resampling procedure (Validation set). The performance of the nomogram was assessed using discrimination and calibration (Validation set). The proposed nomogram’s discrimination ability was quantified with a receiver operating characteristic (ROC) curve analysis and the AUC. The calibration was carried out by plotting the calibration curve to analyze the association between the observed incidence and the predicted probability. Decision curve analysis was performed to assess the clinical utility of the nomogram (Training set and Validation set). Statistical analysis was conducted with R software (version 3.4.3). Statistical significance was defined as ***p*** < 0.05.

## Results

### Comparison of Baseline Patient Characteristics Between SAE and Non-SAE Groups and Between Primary and Validation Cohorts

**FIGURE 1:**
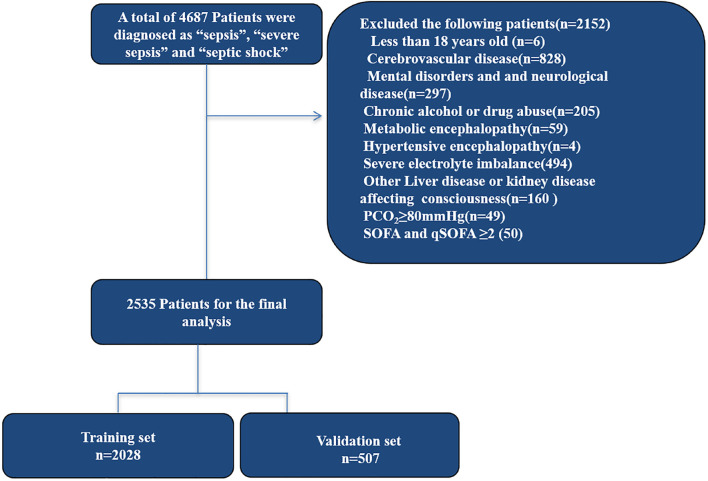
Study design and flow chart of the enrollment process. MIMIC III, Medical Information Mart for Intensive Care III.

A total of 2,535 patients met the inclusion criteria for the study. About 80% of the patients were randomly assigned to the primary cohort, and 20% of the patients were randomly assigned to the validation cohort. About 41.6% of patients with sepsis-associated encephalopathy were detected. A matrix diagram of missing data is shown in the Data Profiling Report (Supplementary Material 12). We replaced any missing values of the included variables with their mean values. The recruitment process is shown in Figure 1.

Patient characteristics in the primary and validation cohorts are given in Table 1. There were no significant differences between the two cohorts in SAE (*P* = 0.763), where the SAE patients were 41.5 and 42.2% in the primary and validation cohorts, respectively, and there were no significant differences in the clinical characteristics between the cohorts, which justified their use as training and validation cohorts.

**TABLE 1:** Baseline characteristics and outcome of patient with SAE.

	Primary cohort			Validation cohort		
	SAE patients, *n* = 841	Non-SAE patients, *n* = 1,187	*P*	SAE patients, *n* = 214	Non-SAE patients, *n* = 293	*P*
Age, (years)	73 (60.9–83.1)	67.8 (54.8–79.1)	<0.001	73.8 (60.8–83.9)	67.1 (55.6–77.7)	0.001
**Sex, n (%)**						
Female	393 (46.7)	509 (42.9)	0.093	112 (52.3)	137 (46.8)	0.242
Male	448 (53.3)	678 (57.1)		91 (42.5)	156 (53.2)	
**Admission_type, n (%)**						
Emergency	51 (6.1)	85 (7.2)	0.622	8 (3.7)	16 (5.5)	0.396
Elective	774 (92.0)	1,080 (91.0)		204 (95.3)	271 (92.5)	
Urgent	16 (1.9)	22 (1.9)		2 (0.9)	6 (2.0)	
**Marital_status, n (%)**						
Married	401 (47.7)	575 (48.4)	0.001	101 (47.2)	135 (46.1)	0.282
Widowed	177 (21.0)	169 (14.2)		44 (20.6)	52 (17.7)	
Single	178 (21.2)	304 (25.6)		40 (18.7)	76 (25.9)	
Divorced	46 (5.5)	66 (5.6)		16 (7.5)	14 (4.8)	
Separated	39 (4.6)	73 (6.1)		10 (4.7)	16 (5.5)	
**Comorbidity, n (%)**						
Hypertension	503 (59.8)	681 (57.4)	0.293	139 (65.0)	167 (57.0)	0.081
Diabetes	254 (30.2)	326 (27.5)	0.195	73 (34.1)	75 (25.6)	0.038
Cardiovascular diseases	562 (66.8)	777 (65.5)	0.536	142 (66.4)	204 (69.6)	0.441
Chronic pulmonary disease	167 (19.9)	260 (21.9)	0.270	51 (23.8)	64 (21.8)	0.592
Liver disease	87 (10.3)	125 (10.5)	0.941	19 (8.9)	39 (13.3)	0.157
Anemias	408 (48.5)	562 (47.3)	0.620	100 (46.7)	131 (44.7)	0.653
Acidosis	222 (26.4)	334 (28.1)	0.391	45 (21.0)	69 (23.5)	0.520
Alkalosis	30 (3.6)	60 (5.1)	0.125	3 (1.4)	15 (5.1)	0.028
Hypovolemia	64 (7.6)	83 (7.0)	0.603	16 (7.5)	18 (6.1)	0.592
**Chronic treatments, n (%)**						
Statins	99 (11.8)	87 (7.3)	0.001	31 (14.5)	21 (7.2)	0.011
Beta blockers	210 (25.0)	136 (11.5)	<0.001	66 (30.8)	32 (10.9)	<0.001
H_2_-antagonist	117 (13.9)	68 (5.7)	<0.001	35 (16.4)	14 (4.8)	<0.001
Proton pump inhibitor	261 (31.0)	252 (21.2)	<0.001	72 (33.6)	58 (19.8)	<0.001
Steroids	207 (24.6)	150 (12.6)	<0.001	56 (26.2)	36 (12.3)	<0.001
NSAIDs	263 (31.3)	209 (17.6)	<0.001	69 (32.2)	48 (16.4)	<0.001
Aspirin	159 (18.9)	121 (10.2)	<0.001	47 (22.0)	30 (10.2)	<0.001
Clopidogrel	56 (6.7)	34 (2.9)	<0.001	11 (5.1)	6 (2.0)	0.078
Sodium bicarbonate	105 (12.5)	111 (9.4)	0.028	30 (14.0)	27 (9.2)	0.117
Vitamin D	29 (3.4)	33 (2.8)	0.433	9 (4.2)	5 (1.7)	0.105

*NSAIDs, non-steroidal anti-inflammatory drugs; SAE, sepsis-associated encephalopathy.*

Patients in the SAE group were older than those in the non-SAE group in the primary cohort and validation cohort. More SAE patients used statins, beta-blockers, H_2_-antagonist, proton pump inhibitor, steroids, non-steroidal anti-inflammatory drugs (NSAIDs), aspirin, clopidogrel, and sodium bicarbonate compared to the non-SAE group. There were no significant differences in gender, admission type, marital status, and comorbidity between the SAE and non-SAE patients.

### Characteristics of Patients on ICU Admission

As shown in Table 2, patients with SAE were more likely to suffer from urinary tract infections. Compared to the non-SAE patients, more SAE patients used antimicrobial drugs, including antiviral drugs, cephalosporins, penicillin, antifungal drugs, macrolides, quinolones, and carbapenem; analgesic and sedative drugs including propofol, midazolam, opioids, and oxycodone; vasopressor; diphenhydramine Hydrochloride; calcium gluconate; magnesium sulfate; and heparin sodium injection. Patients in the SAE group were more critically ill than the non-SAE group [SOFA 6 (4–9) vs. 5 (3–8), *p* < 0.001; qSOFA 2 (2–3) vs. 2 (2–2), *p* < 0.001; SAPS II 45 (35–57) vs. 40 (31–51), *p* < 0.001]. The hospital mortality rate of SAE patients was 64.7%. Patients in the non-SAE group had a higher incidence of mechanical ventilation (53.8 vs. 42.4%) and had longer ICU stay time than those in the SAE group.

**TABLE 2:** Multivariate logistic analysis of risk factors to incidence in patients with SAE.

	Primary cohort			Validation cohort		
	SAE patients *n* = 841	Non-SAE patients, *n* = 1,187	*P*	SAE patients, *n* = 214	Non-SAE patients, *n* = 293	*P*
**Infection site, n (%)**						
Lung	344 (40.9)	476 (40.1)	0.748	82 (38.3)	116 (39.6)	0.783
Intestinal	153 (18.2)	221 (18.6)	0.816	37 (17.3)	47 (16.0)	0.718
Urinary system	283 (33.7)	338 (28.5)	0.014	90 (42.1)	89 (30.4)	0.008
Catheter related	120 (14.3)	149 (12.6)	0.288	39 (18.2)	43 (14.7)	0.903
Skin and soft tissue	113 (13.4)	180 (15.2)	0.305	29 (13.6)	47 (16.0)	0.453
Abdominal cavity	195 (23.2)	182 (15.3)	0.489	48 (22.4)	67 (22.9)	1.000
**Microorganisms, n (%)**						
Gram-positive	433 (51.5)	579 (48.8)	0.241	114 (53.3)	143 (48.8)	0.324
Gram-negative	407 (48.4)	581 (49.0)	0.822	106 (49.5)	121 (41.3)	0.071
Fungus	318 (37.8)	456 (38.4)	0.817	82 (38.3)	103 (35.2)	0.513
Virus	18 (2.1)	33 (2.8)	0.391	4 (1.9)	7 (2.4)	0.767
**Medications, n (%)**						
**Antibiotic**						
Antiviral drug	30 (3.6)	18 (1.5)	0.004	13 (6.1)	4 (1.4)	0.005
Cephalosporins	170 (20.2)	124 (10.4)	<0.001	40 (18.7)	28 (9.6)	0.004
Penicillin	142 (16.9)	92 (7.7)	<0.001	35 (16.4)	23 (7.8)	0.004
Antifungal	172 (20.5)	148 (12.5)	<0.001	50 (23.4)	39 (13.3)	0.004
Macrolides	77 (9.2)	55 (4.6)	<0.001	25 (11.7)	16 (5.5)	0.013
Aminoglycosides	60 (7.1)	38 (3.2)	<0.001	9 (4.2)	8 (2.7)	0.455
Quinolones	234 (27.8)	149 (12.6)	<0.001	70 (32.7)	34 (11.6)	<0.001
Carbapenem antibiotics	86 (10.2)	45 (3.8)	<0.001	25 (11.7)	10 (3.4)	0.001
Sulfamethoxazole	31 (3.7)	46 (3.9)	0.906	16 (7.5)	10 (3.4)	0.065
**Analgesic and sedative drugs**						
Propofol	142 (16.9)	93 (7.8)	<0.001	42 (19.6)	22 (7.5)	<0.001
Midazolam	156 (18.5)	99 (8.3)	<0.001	45 (21.0)	28 (9.6)	<0.001
Opioids	273 (32.5)	213 (17.9)	<0.001	76 (35.5)	53 (18.1)	<0.001
Etomidate	12 (1.4)	14 (1.2)	0.690	9 (4.2)	2 (0.7)	0.011
Oxycodone	119 (14.1)	121 (10.2)	0.008	35 (16.4)	26 (8.9)	0.013
Vasopressor	245 (29.1)	225 (19.0)	<0.001	63 (29.4)	47 (16.0)	<0.001
Diphenhydramine Hydrochloride	90 (10.7)	47 (4.0)	<0.001	15 (7.0)	10 (3.4)	0.095
Metoclopramide	68 (8.1)	76 (6.4)	0.160	20 (9.3)	22 (7.5)	0.515
**Electrolyte solution**						
Calcium gluconate	231 (27.5)	171 (14.4)	<0.001	61 (28.5)	41 (14,0)	<0.001
Magnesium Sulfate	208 (24.7)	248 (20.9)	0.046	66 (30.8)	58 (19.8)	0.005
Thiamine	20 (2.4)	34 (2.9)	0.487	11 (5.1)	5 (1.7)	0.038
Heparin Sodium Injection	258 (30.7)	228 (19.2)	<0.001	79 (36.9)	54 (18.4)	<0.001
**Vital signs**						
Heart rate (bmp)	110 (94–126)	111 (95–127)	0.768	113 (98–129)	112 (95–130)	0.895
Dys bp (mmHg)	82 (73–92)	84 (75–93)	0.032	84 (74–94)	83 (75–91)	0.810
Dias bp (mmHg)	38 (31–46)	41 (34–47)	<0.001	39 (31.8–46)	42 (33.5–49)	0.007
Resp rate (bmp)	28 (25–33)	29 (25–34)	0.226	29 (24–33.3)	29 (24.5–34)	0.460
Tempc (°C)	37.4 (36.9–38.2)	37.5 (37.0–38.3)	0.220	37.6 (37.0–38.2)	37.6 (37.0–38.3)	0.929
**Laboratory parameters**						
Lactate (mmol/L)	1.9 (1.3–3.0)	1.9 (1.3–2.9)	0.494	1.9 (1.4–3.1)	1.8 (1.3–3.0)	0.294
PCO_2_ (mmHg)	40 (35–43)	40 (35–43)	0.766	40 (35–42)	40 (34–44)	0.753
PO_2_ (mmHg)	92 (89–95)	92 (89–95)	0.101	92 (89–94.3)	92 (90–95)	0.387
PH	7.34 (7.31–7.40)	7.34 (7.30–7.40)	0.725	7.34 (7.33–7.39)	7.34 (7.30–7.39)	0.425
Glucose (mg/dL)	113 (96–135)	114 (97–135)	0.497	114.5 (99–140)	115 (97.5–137)	0.694
Creatinine (mg/dL)	1.2 (0.8–2.1)	1.2 (0.8–2.1)	0.133	1.2 (0.7–2.0)	1.2 (0.8–2.1)	0.335
Blood urea nitrogen (mg/dL)	26 (17–44)	25 (15–43)	0.091	26 (15–38)	27 (17–43)	0.088
Alanine aminotransferase (IU/L)	27 (15–53.5)	27 (16–65)	0.025	27 (14–57)	27 (16–58)	0.408
Aspartate aminotransferase (IU/L)	35 (22–66)	34 (21–71)	0.799	31 (21–64.3)	35 (21–66)	0.244
Albumin (g/dL)	2.8 (2.4–3.2)	2.9 (2.4–3.3)	0.359	2.9 (2.5–3.4)	2.8 (2.4–3.2)	0.096
Hemoglobin (g/dL)	9.7 (8.6–10.9)	9.6 (8.6–10.8)	0.265	9.5 (8.4–10.6)	9.6 (8.3–10.8)	0.586
Platelet (K/uL)	179 (117–257)	181 (111–261)	0.842	174.5 (112–278.3)	192 (116–279)	0.422
Potassium (mEq/L)	4.1 (3.7–4.5)	4.0 (3.7–4.4)	0.499	4.0 (3.7–4.6)	4.0 (3.7–4.5)	0.609
Sodium (mEq/L)	139 (136–142)	138 (135–141)	0.003	139 (136–142)	139 (135.5–141)	0.599
Partial thromboplastin time (s)	31.9 (27.7–38.7)	32.4 (27.9–39.7)	0.508	30.5 (26.4–38.5)	30.9 (27.7–38.0)	0.331
White blood cell count (K/uL)	10.9 (7.2–15.3)	11.3 (7.2–16.9)	0.203	11.2 (7.9–16.8)	11.8 (7.2–17.4)	0.971
Lymphocyte (%)	9.4 (5.2–16.0)	8.9 (4.4–16.0)	0.103	10.0 (5.6–16.1)	8.6 (4.9–14)	0.028
Neutrophil (%)	80.7 (70.2–87.8)	80 (70–87.9)	0.775	79.7 (69.0–87.1)	83 (73.9–88.3)	0.021
Monocytes (%)	4.0 (2.5–6.0)	4.0 (2.4–6.0)	0.732	4.0 (2.4–6.3)	4.0 (2.7–5.4)	0.445
Eosinophils (%)	0.4 (0.0–1.8)	0.4 (0.0–1.6)	0.579	0.6 (0.0–1.8)	0.6 (0.0–1.6)	0.728
**Outcome**						
Mechanical ventilation, n (%)	357 (42.4)	639 (53.8)	<0.001	81 (37.9)	155 (52.9)	0.001
Renal replacement therapy, n (%)	49 (5.8)	90 (7.6)	0.130	8 (3.8)	23 (7.8)	0.062
**Organ failure, n (%)**						
Respiratory	371 (44.1)	584 (49.2)	0.024	89 (41.6)	149 (50.9)	0.047
Cardiovascular	408 (48.5)	591 (49.8)	0.589	99 (46.3)	137 (46.8)	0.928
Renal	504 (59.9)	685 (57.7)	0.337	133 (62.1)	175 (59.7)	0.645
Hepatic	56 (6.7)	89 (7.5)	0.485	13 (6.1)	23 (7.8)	0.488
Hematologic	198 (23.5)	237 (20.0)	0.055	51 (23.8)	59 (20.1)	0.328
SOFA	6.0 (4.0–9.0)	5.0 (3.0–8.0)	<0.001	6.0 (3.0–9.0)	5.0 (3.0–8.0)	0.199
qSOFA	2.0 (2.0–3.0)	2.0 (2.0–2.0)	<0.001	2.0 (2.0–3.0)	2.0 (2.0–2.0)	<0.001
SAPS II	45 (35–57)	40 (31–51)	<0.001	44 (33–57)	40 (32–53)	0.014
ICU stay time, days	2.9 (1.7–6.8)	3.4 (1.8–8.7)	0.010	2.5 (1.5–5.7)	3.3 (1.7–9.2)	0.015
Hospital mortality, n (%)	258 (30.7)	318 (26.8)	0.056	64 (30.0)	92 (31.4)	0.719

### Feature Selection and SAE Signature Building

**FIGURE 2:**
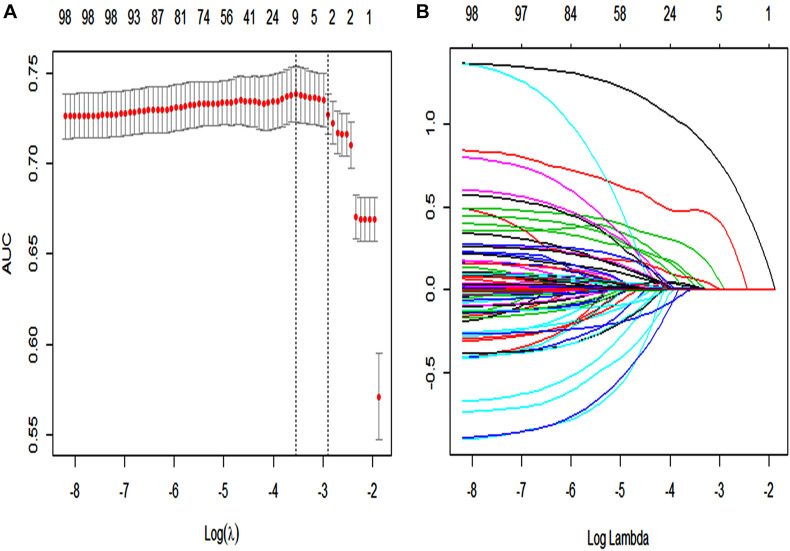
The LASSO logistic regression model was used by texture feature selection. **(A)** 10-fold cross-validation *via* minimum criteria was used by tuning parameter (λ) selection in the LASSO model. The area under the receiver operating characteristic curve (AUC) was plotted vs. log(λ). For each λ value, around the mean value of the target parameter shown by the red dot, we can get a confidence interval for the target parameter. The two dashed lines, respectively, indicate two special values of λ. The dotted line on the left in panel **(A)** (lambda.min) refers to the one that obtains the mean value of the smallest target parameter among all λ values. The dotted line on the right in panel **(A)** (lambda.1se) refers to the λ value of the simplest model within a variance range of lambda.min. A λ value of 0.009, with log(λ) = 24.709 was chosen (1-SE criteria) according to 10-fold cross-validation. **(B)** LASSO coefficient profiles of the 89 texture features. Each curve in the panel **(B)** represents the change trajectory of each variable coefficient. The ordinate is the value of the coefficient, the lower abscissa is log(λ), and the upper abscissa is the number of non-zero coefficients in the model at this time.

In the primary cohort (Figure 2), 89 features were reduced to nine potential predictors of texture features of 2,028 patients. These features are shown in the SAE-score calculation formula (Supplementary Material 13; Data supplement).

### Development of an Individualized Prediction Model

**FIGURE 3:**
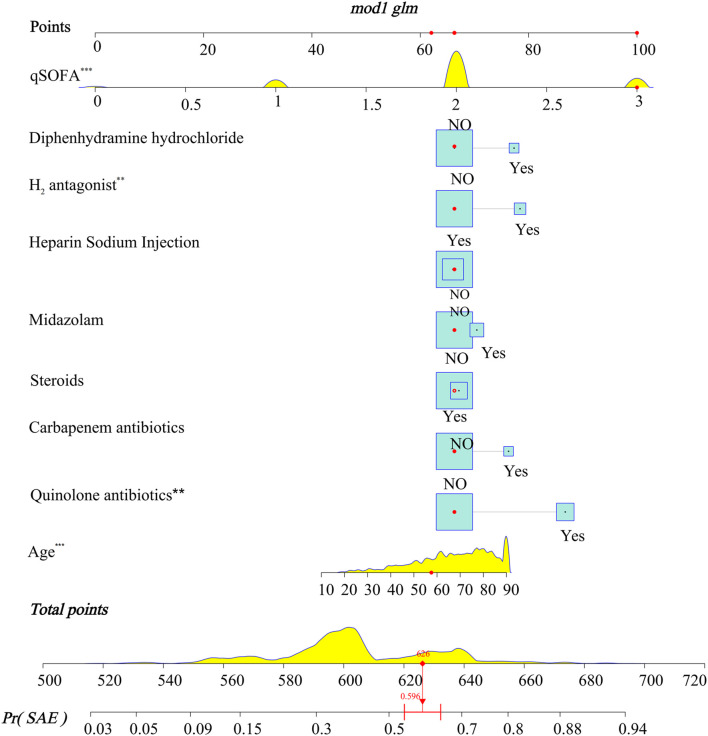
Nomograms for the prediction of the incidence of SAE in patients with sepsis. To use the model, firstly, the position of each variable on the corresponding axis should be determined. Secondly, draw a line to the points axis for the number of points, and then add the points from all the variables. Thirdly, draw a line from the total points axis to determine the incidence of SAE at the lower line of the nomogram. The total points projected to the bottom scale indicate the percentage probability of the incidence of SAE. *** < 0.001, ** < 0.05.

A LASSO logistic regression analysis identified age, qSOFA, quinolone antibiotics, carbapenem antibiotics, midazolam, diphenhydramine hydrochloride, heparin sodium injection, steroids, and H_2_-antagonist as independent predictors (Supplementary Material 13). The nomogram included all the significant independent factors of the logistic regression model in the training cohort. It established scoring criteria according to the odds ratio (OR) values of risk factors and gave a score for each level of prognostic factors. Through summation of the scores associated with each variable and projection of the total scores to the bottom scale, probabilities could be estimated for SAE, and it was possible to effectively predict SAE according to the individual characteristics of the patient. The diagnostic nomogram for SAE is shown in Figure 3.

### Discrimination and Calibration

**FIGURE 4:**
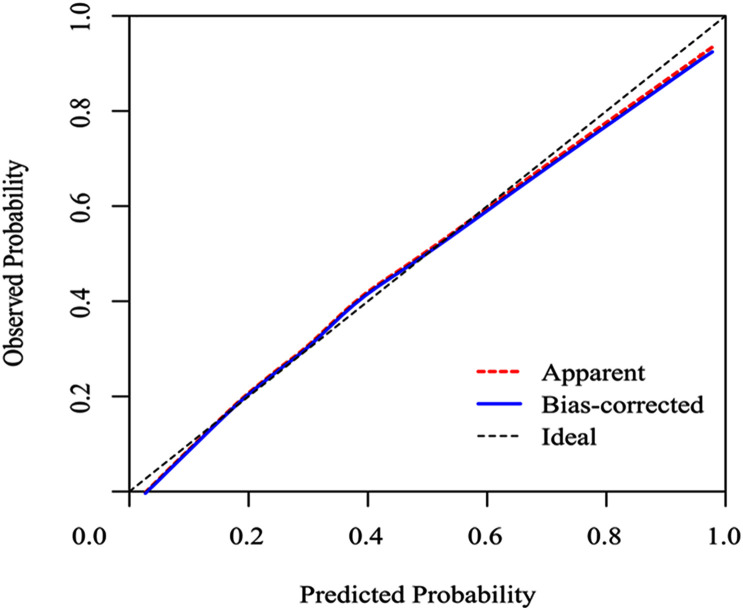
Calibration curves of a nomogram estimating the incidence of SAE in sepsis patients. Predicted and observed SAE rates are plotted as the logistic calibration. The y-axis represents the actual SAE occurrence rate. The x-axis represents the predicted SAE occurrence risk. The diagonal dotted line represents a perfect prediction by an ideal model. The blue solid line represents the performance of the nomogram, of which a closer fit to the diagonal dotted line represents a better prediction.

To evaluate the calibration of the model, the study used internal validation with the 1,000 bootstrap resampling method as shown in Figure 4. The calibration plot of current depression rates suggests good agreement between the observed and predicted values.

**FIGURE 5:**
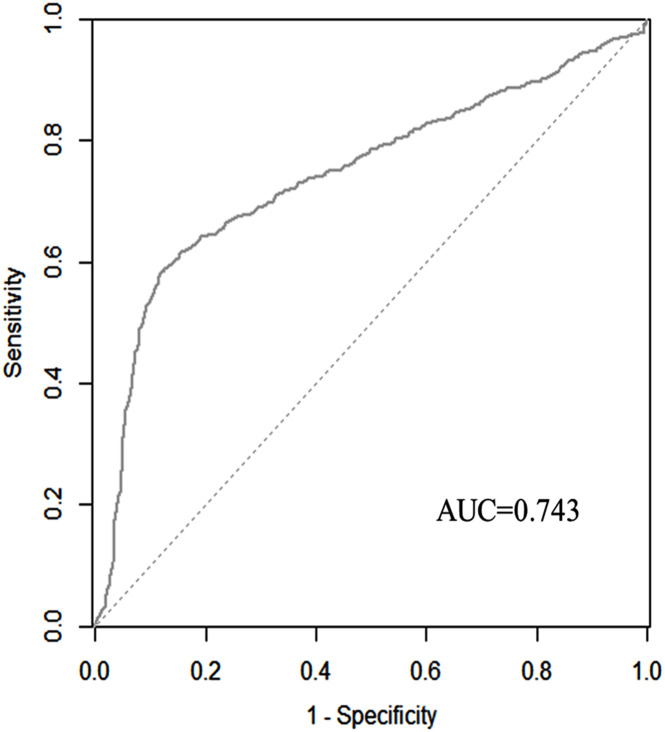
Discriminatory accuracy for predicting the incidence of SAE assessed by receiver operator characteristics (ROC) analysis calculating area under the curve (AUC).

We used the ROC curve to evaluate the discrimination capability of the model. The area under the curve (AUC) of the nomogram was 0.743 (95% CI: 0.720–0.766). The predictive SAE of the model’s sensitivity was 0.585 and specificity was 0.879. A cut-off was 0.435 calculated by Youden’s index (Figure 5).

### Clinical Utility

**FIGURE 6:**
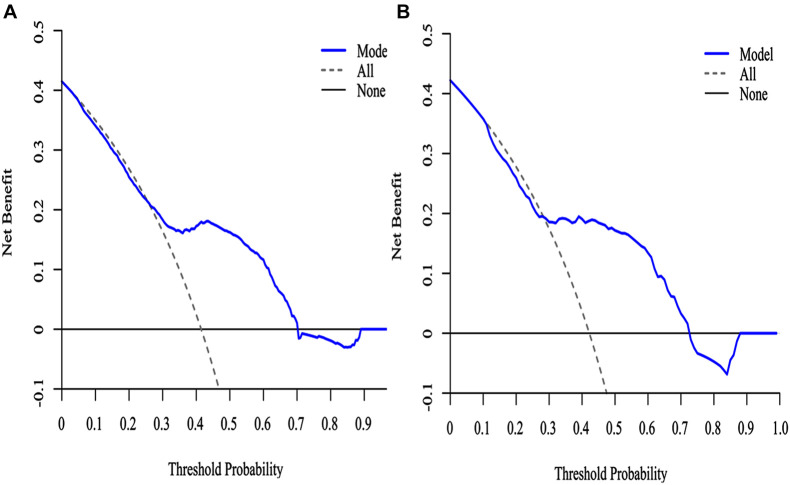
The DCA curve of SAE patients with the nomogram. **(A)** Primary cohort; **(B)** validation cohort. Solid line: The patient does not use the predictive SAE model without treatment for SAE, the net benefit is zero; Gray line: All patients used the predictive SAE model with effective treatment measures; Blue lines: If the SAE predictive model exceeds a threshold (ranging from approximately 10–90%), the patient needs to be treated immediately. For example, a patient would be treated for SAE if the probability was greater than 10%.

The decision curve analysis (DCA) for the SAE nomogram is in Figure 6. According to the DCA, the SAE model of net benefit had threshold probabilities ranging from 10 to 90% in the primary cohort. The decision curve showed that if the threshold probability was between 10% and 90%, using the SAE nomogram to predict SAE would be of more benefit to predict SAE compared to not utilizing the nomogram. In the validation set, the SAE model of net benefit had threshold probabilities ranging from 10 to 89%, and thus the model was beneficial in its prediction of SAE.

## Discussion

The study observed that 41.6% of sepsis patients with SAE were identified during admission to the ICU. A hospital mortality rate of up to 64.7% was observed in patients with SAE. We identified that clinically relevant risk factors for SAE, including age, qSOFA score, and medications such as carbapenem antibiotics, quinolones antibiotics, steroids, midazolam, H_2_-antagonist, diphenhydramine hydrochloride, and heparin sodium injection, had a significant impact on the occurrence of SAE. The study has established a comprehensive visual prediction model which can provide a probabilistic estimate of SAE at the earlier stages in individual sepsis patients. Furthermore, the nomogram showed satisfactory validity, discrimination, and clinical utility.

In this study, 41.6% of sepsis patients suffered from SAE. Previous studies have published the rates of SAE in patients with sepsis ranging from 8 to 70% (Bartynski et al., 2006). This could be the result of different diagnostic criteria. Feng et al. (2019) reported a 42.3% incidence of SAE in septic patients, whereas Yang et al. (2020) reported 50% incidence. This study result is consistent with their study results.

Our cohort study showed that SAE patients had higher SOFA, qSOFA, and APACHE II when compared to non-SAE patients and also a high hospital mortality rate of 64.7%. It shows that SAE patients with more severe organ dysfunction are associated with an increased risk of mortality and the related adverse clinical outcomes. The result is consistent with Yang et al.’s study. SAE patients presented significantly high APACHE II, SOFA scores, and 30-day mortality in a recent retrospective analysis involving more than 2,400 SAE patients (Yang et al., 2020). Feng et al. (2019) demonstrated that the incidence of 28-day mortality was 45.95% and 180-day mortality was 55.41%, and the multivariate stepwise regression analysis demonstrated that the risk of death in the SAE group was significantly higher than in the non-SAE group and that SAE was a risk factor for sepsis-related death (OR = 2.868). These results are consistent with our findings.

We identified clinical and potential risk factors for SAE, which confirms that SAE patients were older and had urinary system infection when compared to non-SAE patients. Sonneville et al. (2017) showed that compared to the non-SAE group, the SAE group included patients who were significantly older in age. Presence of comorbid urinary system infection in patients with delirium had been confirmed by many studies (Chae and Miller, 2015; Carson et al., 2017). Previous studies have also reported that urinary system infection is a risk factor for delirium (Gau et al., 2009; Dahl et al., 2010), urinary tract infections (UTI) increase a subject’s risk of developing delirium, or urinary system infection is a “common cause” of delirium (Lerner et al., 1997; Kamel, 2005). The potential mechanisms of the association between urinary system infection and neuropsychiatric disorders are induced by antibiotic treatment for urinary system infection. The antibiotics that were most frequently implicated were macrolides and fluoroquinolones. Mostafa et al. reported 15 cases of antibiotic-induced psychosis during treatment of a urinary system infection, with 60% of the cases determined to be “highly suggestive” of a causal relationship between antibiotic usage and psychosis (Mostafa and Miller, 2014). Contrary to previously published data (Zhang et al., 2012; Sonneville et al., 2017), we did not find any microorganism as the pathogen associated with SAE. It is attributed to different data sources.

The results of our cohort study show that medications were a critical risk factor for SAE. The most significant impact is from the use of antibiotics, followed by analgesics, sedative drugs, and other drugs. The study demonstrated that more SAE patients used antivirals, cephalosporins, penicillin, antifungal, macrolides, aminoglycosides, quinolones, carbapenem in antibiotic, quinolones, and carbapenem are associated with SAE by filtervariables with the LASSO method. This method surpasses the method of choosing predictors based on the strength of their univariable association with the outcome. In the study, the use of quinolones and carbapenem tended to be associated with SAE, in line with the study by Sonneville et al. (2017) and recent reviews highlighting their neurotoxicity (Bhattacharyya et al., 2016). Previous studies have suggested that the pathophysiology of quinolones and carbapenem-associated encephalopathy is associated with a disturbance of gamma-aminobutyric acid-ergic (GABAergic) interneurons. They affect the central nervous system mainly by inhibiting GABA receptors interfering with inhibitory neurotransmission and enhancing bursts of excitatory neurons, which is concentration-dependent (Hori and Shimada, 1993; Munoz-Gomez et al., 2015). Our results also suggested that the use of propofol, midazolam, and opioids was found to be more likely observed in SAE patients. Further analysis found that midazolam is found to be a risk factor with SAE patients. It is widely accepted that midazolam is an independent risk factor for delirium in critically ill patients. In a large population-based cohort study, Zaal et al. found that the risk of delirium occurrence in critically ill adults is related to benzodiazepine use. A daily dose of only 5 mg of midazolam administered to a coma- and delirium-free patient increased the odds of this patient developing delirium in the following day by 4%. It supports the study results. A large number of SAE patients used vasopressors. On the one hand, SAE patients were found to be more critically ill and had a higher incidence of circulatory failure. On the other hand, the use of vasopressor was found to be a risk factor for SAE, although our further analysis has not been confirmed. Vasopressor use is a known risk factor for long-term cognitive impairment after critical illness. However, the specific mechanisms of these factors cannot be individually determined by our data, and this question requires further research.

Besides, our cohort study demonstrated that the use of H_2_-antagonist, steroids, and heparin sodium injection were risk factors for SAE. It has been reported by many studies that H_2_-antagonist and steroids cause delirium (Nguyen et al., 2011; Mauran et al., 2016). Tawadrous et al. (2014) demonstrated that compared to a lower dose, initiation of the current standard dose of histamine 2 receptor antagonists (H2RA) in older adults is associated with a small absolute increase in the 30-day risk of altered mental status. Yamada et al. (2018) found that steroid use was the determinant of progression to delirium in an intensive care unit, and research by Romain Sonneville et al. also found that steroid use was an independent risk factor for SAE (Yamada et al., 2018). These studies supported our study results. An interesting finding in the study was that heparin sodium injection and diphenhydramine hydrochloride were found to be risk factors for SAE. The result is consistent with several other studies (Shobugawa et al., 2007; Rothberg et al., 2013; Bidaki et al., 2017). However, the study for the first time demonstrated a strong association between heparin sodium injection, diphenhydramine hydrochloride, and SAE in a large cohort of sepsis patients. It is proposed that we pay attention to monitoring the mental changes in patients when using the above drugs.

The lack of validated predictive tools for early-stage SAE in sepsis patients and the equivocal efficacy of SAE interventions prompted us to develop a novel predictive modeling system using the nomogram methodology. For the construction of the clinical features and risk factors, 89 candidate features were reduced to nine potential predictors (carbapenem antibiotics, quinolones antibiotics, steroids, midazolam, H_2_-antagonist, diphenhydramine hydrochloride, and heparin sodium injection) by the LASSO method. The nine potential predictors established a comprehensive visual nomogram for predicting SAE patients. The nomogram demonstrated adequate discrimination in the primary cohort (AUC, 0.743; 95% CI: 0.720–0.766), which surprisingly improved in the validation cohort (AUC, 0.762; 95% CI: 0.716–0.807). We developed and validated a nomogram in the study, which could assess clinical variables. Both physicians and patients could perform an individualized prediction of the risk of SAE with this easy-to-use scoring system, which is in line with the current trend toward personalized medicine. The most important reason for using the nomogram is based on the need to interpret the individualized needs for additional treatment and improve patient outcomes. DCA was applied in this study. This novel method offers insight into the clinical consequences based on the threshold probability, from which the net benefit could be derived. The decision curve showed that if the threshold probability of a patient or doctor was > 10%, then using the SAE nomogram to predict SAE added more benefit compared to not using the SAE nomogram.

There were two limitations in the study. Firstly, the study was based on electronic MIMIC-III, whose data were generated during routine clinical practice. Thus, it is possible that the cohort selection is not exactly consistent with the definition of sepsis based on the guidelines, and neuroimaging data were also not included in the database. Besides, the study only conducted internal validation, and thus external validations are needed. Thus, the current nomogram can only provide a certain reference for SAE forecasts, and further modifications may be required once diagnostic methods are developed.

## Conclusion

A nomogram was established for individualized prediction of SAE in sepsis patients and it showed satisfactory performance. It can be conveniently used in the clinical setting and may help physicians to identify SAE patients on time. It can also help physicians to take timely intervention measures to reduce the incidence of SAE and improve patient prognosis.

## Data Availability Statement

The original contributions presented in the study are included in the article/Supplementary Material, further inquiries can be directed to the corresponding author/s.

## Ethics Statement

Ethical review and approval was not required for the study on human participants in accordance with the local legislation and institutional requirements. Written informed consent from the patients/participants or patients/participants’ legal guardian/next of kin was not required to participate in this study in accordance with the national legislation and the institutional requirements.

## Author Contributions

LZ and YW developed the central ideas for this work and wrote the first draft of the manuscript. HZ and ZG collected the data regarding the manuscript. YL revised the manuscript, worked on the English, and made the final version of the manuscript. All authors read and approved the final manuscript.

## Conflict of Interest

The authors declare that the research was conducted in the absence of any commercial or financial relationships that could be construed as a potential conflict of interest.

## Publisher’s Note

All claims expressed in this article are solely those of the authors and do not necessarily represent those of their affiliated organizations, or those of the publisher, the editors and the reviewers. Any product that may be evaluated in this article, or claim that may be made by its manufacturer, is not guaranteed or endorsed by the publisher.

## Supplementary Material

Supplementary Material 1 Exclude patients with trauma of skull from the MIMIC-III database according to ICD9-codes.

Supplementary Material 2 Exclude patients with intracerebral hemorrhage, cerebral embolism, and ischemic stroke disease from the MIMIC-III database according to ICD9-codes.

Supplementary Material 3 Exclude patients with meningitis and encephalitis disease from the MIMIC-III database according to ICD9-codes.

Supplementary Material 4 Exclude patients with Epilepsy disease from the MIMIC-III database according to ICD9-codes.

Supplementary Material 5 Exclude patients with other cerebrovascular disease from the MIMIC-III database according to ICD9-codes.

Supplementary Material 6 Exclude patients with mental disorders and neurological disease from the MIMIC-III database according to ICD9-codes.

Supplementary Material 7 Exclude patients with alcoholic intoxication or drug abuse from the MIMIC-III database according to ICD9-codes.

Supplementary Material 8 Exclude patients with metabolic encephalopathy, hepatic encephalopathy, hypertensive encephalopathy, diabetes with coma, disorders of urea cycle, and hypernatremia from the MIMIC-III database according to ICD9-codes.

Supplementary Material 9 Type of disease and ICD9-codes.

Supplementary Material 10 Infection site and ICD9-code.

Supplementary Material 11 Organ failure was defined by a combination of ICD-9-CM and CPT codes.

Supplementary Material 12 Data profiling report.

Supplementary Material 13 Feature Selection, data supplement.

## Abbreviations

SAE: sepsis-associated encephalopathy
SAPS II: simplified acute physiology score
SOFA: sequential organ failure assessment
qSOFA: quick sequential organ failure assessment
GCS: Glasgow coma scale
ICU: intensive care unit
MIMIC III: Medical Information Mart for Intensive Care III
ICD-9, International Classification of Diseases: Ninth Revision
ROC: Receiver operating characteristic curve.
