# Supplementary material for: Mechanical Learning for Prediction of Sepsis-Associated Encephalopathy
Source: Front Comput Neurosci. 2021 Nov 16;15:739265. doi: 10.3389/fncom.2021.739265 (PMC8636425; doi:10.3389/fncom.2021.739265)
Supplement: Supplementary Material 1 — Exclude patients with trauma of skull from the MIMIC-III database according to ICD9-codes. [file Data_Sheet_1.zip › Supplementary materials/Supplementary Material 1.docx]

| **Supplementary materials1** Exclude patients with trauma of skull from the MIMIC III database according to ICD9-codes | | |
| --- | --- | --- |
| ICD9-code |  | Description |
| 80016 |  | Closed fracture of vault of skull with cerebral laceration and contusion,  with loss of consciousness of unspecified duration |
| 80019 |  | Closed fracture of vault of skull with cerebral laceration and contusion,  with concussion, unspecified |
| 80020 |  | Closed fracture of vault of skull with subarachnoid, subdural, and extradural hemorrhage,  unspecified state of consciousness |
| 80021 |  | Closed fracture of vault of skull with subarachnoid, subdural, and extradural hemorrhage,  with no loss of consciousness |
| 80022 |  | Closed fracture of vault of skull with subarachnoid, subdural, and extradural hemorrhage,  with brief [less than one hour] loss of consciousness |
| 80023 |  | Closed fracture of vault of skull with subarachnoid, subdural, and extradural hemorrhage,  with moderate [1-24 hours] loss of consciousness |
| 80024 |  | Closed fracture of vault of skull with subarachnoid, subdural, and extradural hemorrhage,  with prolonged [more than 24 hours] loss of consciousness and return to pre-existing conscious level |
| 80025 |  | Closed fracture of vault of skull with subarachnoid, subdural, and extradural hemorrhage,  with prolonged [more than 24 hours] loss of consciousness, without return to pre-existing conscious level |
| 80026 |  | Closed fracture of vault of skull with subarachnoid, subdural, and extradural hemorrhage,  with loss of consciousness of unspecified duration |
| 80029 |  | Closed fracture of vault of skull with subarachnoid, subdural, and extradural hemorrhage,  with concussion, unspecified |
| 80030 |  | Closed fracture of vault of skull with other and unspecified intracranial hemorrhage,  unspecified state of consciousness |
| 831 |  | Closed fracture of vault of skull with other and unspecified intracranial hemorrhage,  with no loss of consciousness |
| 832 |  | Closed fracture of vault of skull with other and unspecified intracranial hemorrhage,  with brief [less than one hour] loss of consciousness |
| 833 |  | Closed fracture of vault of skull with other and unspecified intracranial hemorrhage,  with moderate [1-24 hours] loss of consciousness |
| 834 |  | Closed fracture of vault of skull with other and unspecified intracranial hemorrhage, with prolonged [more than 24 hours] loss of consciousness and return to pre-existing conscious level |
| 835 |  | Closed fracture of vault of skull with other and unspecified intracranial hemorrhage, with prolonged [more than 24 hours] loss of consciousness, without return to pre-existing conscious level |
| 836 |  | Closed fracture of vault of skull with other and unspecified intracranial hemorrhage, with loss of consciousness of unspecified duration |
| 8129 |  | Closed fracture of base of skull with subarachnoid, subdural, and extradural hemorrhage, with concussion, unspecified |
| 813 |  | Closed fracture of base of skull with other and unspecified intracranial hemorrhage, unspecified state of consciousness |
| 8131 |  | Closed fracture of base of skull with other and unspecified intracranial hemorrhage, with no loss of consciousness |
| 8132 |  | Closed fracture of base of skull with other and unspecified intracranial hemorrhage, with brief [less than one hour] loss of consciousness |
| 8133 |  | Closed fracture of base of skull with other and unspecified intracranial hemorrhage, with moderate [1-24 hours] loss of consciousness |
| 8134 |  | Closed fracture of base of skull with other and unspecified intracranial hemorrhage, with prolonged [more than 24 hours] loss of consciousness and return to pre-existing conscious level |
| 8135 |  | Closed fracture of base of skull with other and unspecified intracranial hemorrhage, with prolonged [more than 24 hours] loss of consciousness, without return to pre-existing conscious level |
| 8136 |  | Closed fracture of base of skull with other and unspecified intracranial hemorrhage, with loss of consciousness of unspecified duration |
| 8139 |  | Closed fracture of base of skull with other and unspecified intracranial hemorrhage, with concussion, unspecified |
| 814 |  | Closed fracture of base of skull with intracranial injury of other and unspecified nature, unspecified state of consciousness |
| 8141 |  | Closed fracture of base of skull with intracranial injury of other and unspecified nature, with no loss of consciousness |
| 8142 |  | Closed fracture of base of skull with intracranial injury of other and unspecified nature, with brief [less than one hour] loss of consciousness |
| 8143 |  | Closed fracture of base of skull with intracranial injury of other and unspecified nature, with moderate [1-24 hours] loss of consciousness |
| 8144 |  | Closed fracture of base of skull with intracranial injury of other and unspecified nature, with prolonged [more than 24 hours) loss of consciousness and return to pre-existing conscious level |
| 8145 |  | Closed fracture of base of skull with intracranial injury of other and unspecified nature, with prolonged [more than 24 hours] loss of consciousness, without return to pre-existing conscious level |
| 8146 |  | Closed fracture of base of skull with intracranial injury of other and unspecified nature, with loss of consciousness of unspecified duration |
| 8149 |  | Closed fracture of base of skull with intracranial injury of other and unspecified nature, with concussion, unspecified |
| 815 |  | Open fracture of base of skull without mention of intracranial injury, unspecified state of consciousness |
| 8151 |  | Open fracture of base of skull without mention of intracranial injury, with no loss of consciousness |
| 8152 |  | Open fracture of base of skull without mention of intracranial injury, with brief [less than one hour] loss of consciousness |
| 8153 |  | Open fracture of base of skull without mention of intracranial injury, with moderate [1-24 hours] loss of consciousness |
| 8154 |  | Open fracture of base of skull without mention of intracranial injury, with prolonged [more than 24 hours] loss of consciousness and return to pre-existing conscious level |
| 8155 |  | Open fracture of base of skull without mention of intracranial injury, with prolonged [more than 24 hours] loss of consciousness, without return to pre-existing conscious level |
| 8156 |  | Open fracture of base of skull without mention of intracranial injury, with loss of consciousness of unspecified duration |
| 8159 |  | Open fracture of base of skull without mention of intracranial injury, with concussion, unspecified |
| 816 |  | Open fracture of base of skull with cerebral laceration and contusion, unspecified state of consciousness |
| 8161 |  | Open fracture of base of skull with cerebral laceration and contusion, with no loss of consciousness |
| 8162 |  | Open fracture of base of skull with cerebral laceration and contusion, with brief [less than one hour] loss of consciousness |
| 8163 |  | Open fracture of base of skull with cerebral laceration and contusion, with moderate [1-24 hours] loss of consciousness |
| 8164 |  | Open fracture of base of skull with cerebral laceration and contusion, with prolonged [more than 24 hours] loss of consciousness and return to pre-existing conscious level |
| 8165 |  | Open fracture of base of skull with cerebral laceration and contusion, with prolonged [more than 24 hours] loss of consciousness, without return to pre-existing conscious level |
| 8166 |  | Open fracture of base of skull with cerebral laceration and contusion, with loss of consciousness of unspecified duration |
| 8169 |  | Open fracture of base of skull with cerebral laceration and contusion, with concussion, unspecified |
| 817 |  | Open fracture of base of skull with subarachnoid, subdural, and extradural hemorrhage, unspecified state of consciousness |
| 8171 |  | Open fracture of base of skull with subarachnoid, subdural, and extradural hemorrhage, with no loss of consciousness |
| 8172 |  | Open fracture of base of skull with subarachnoid, subdural, and extradural hemorrhage, with brief [less than one hour] loss of consciousness |
| 8173 |  | Open fracture of base of skull with subarachnoid, subdural, and extradural hemorrhage, with moderate [1-24 hours] loss of consciousness |
| 85154 |  | Cerebellar or brain stem contusion with open intracranial wound, with prolonged [more than 24 hours] loss of consciousness and return |
| 85155 |  | Cerebellar or brain stem contusion with open intracranial wound, with prolonged [more than 24 hours] loss of consciousness without return to pre-existing conscious level |
| 85156 |  | Cerebellar or brain stem contusion with open intracranial wound, with loss of consciousness of unspecified duration |
| 85159 |  | Cerebellar or brain stem contusion with open intracranial wound, with concussion, unspecified |
| 8516 |  | Cerebellar or brain stem laceration without mention of open intracranial wound, unspecified state of consciousness |
| 85161 |  | Cerebellar or brain stem laceration without mention of open intracranial wound, with no loss of consciousness |
| 85162 |  | Cerebellar or brain stem laceration without mention of open intracranial wound, with brief [less than 1 hour] loss of consciousness |
| 85163 |  | Cerebellar or brain stem laceration without mention of open intracranial wound, with moderate [1-24 hours] loss of consciousness |
| 85164 |  | Cerebellar or brain stem laceration without mention of open intracranial wound, with prolonged [more than 24 hours] loss of consciousness and return to pre-existing conscious level |
| 85165 |  | Cerebellar or brain stem laceration without mention of open intracranial wound, with prolonged [more than 24 hours] loss of consciousness without return to pre-existing conscious level |
| 85166 |  | Cerebellar or brain stem laceration without mention of open intracranial wound, with loss of consciousness of unspecified duration |
| 85169 |  | Cerebellar or brain stem laceration without mention of open intracranial wound, with concussion, unspecified |
| 8517 |  | Cerebellar or brain stem laceration with open intracranial wound, unspecified state of consciousness |
| 85171 |  | Cerebellar or brain stem laceration with open intracranial wound, with no loss of consciousness |
| 85172 |  | Cerebellar or brain stem laceration with open intracranial wound, with brief [less than one hour] loss of consciousness |
| 85173 |  | Cerebellar or brain stem laceration with open intracranial wound, with moderate [1-24 hours] loss of consciousness |
| 85174 |  | Cerebellar or brain stem laceration with open intracranial wound, with prolonged [more than 24 hours] loss of consciousness and return to pre-existing conscious level |
| 85175 |  | Cerebellar or brain stem laceration with open intracranial wound, with prolonged [more than 24 hours] loss of consciousness without return to pre-existing conscious level |
| 85176 |  | Cerebellar or brain stem laceration with open intracranial wound, with loss of consciousness of unspecified duration |
| 85179 |  | Cerebellar or brain stem laceration with open intracranial wound, with concussion, unspecified |
| 8518 |  | Other and unspecified cerebral laceration and contusion, without mention of open intracranial wound, unspecified state of consciousness |
| 85181 |  | Other and unspecified cerebral laceration and contusion, without mention of open intracranial wound, with no loss of consciousness |
| 85182 |  | Other and unspecified cerebral laceration and contusion, without mention of open intracranial wound, with brief [less than one hour] loss of consciousness |
| 85183 |  | Other and unspecified cerebral laceration and contusion, without mention of open intracranial wound, with moderate [1-24 hours] loss of consciousness |
| 85184 |  | Other and unspecified cerebral laceration and contusion, without mention of open intracranial wound, with prolonged [more than 24 hours] loss of consciousness and return to pre- existing conscious level |
| 85185 |  | Other and unspecified cerebral laceration and contusion, without mention of open intracranial wound, with prolonged [more than 24 hours] loss of consciousness without return to pre-existing conscious level |
| 85186 |  | Other and unspecified cerebral laceration and contusion, without mention of open intracranial wound, with loss of consciousness of unspecified duration |
| 85189 |  | Other and unspecified cerebral laceration and contusion, without mention of open intracranial wound, with concussion, unspecified |
| 8519 |  | Other and unspecified cerebral laceration and contusion, with open intracranial wound, unspecified state of consciousness |
| 85191 |  | Other and unspecified cerebral laceration and contusion, with open intracranial wound, with no loss of consciousness |
| 85192 |  | Other and unspecified cerebral laceration and contusion, with open intracranial wound, with brief [less than one hour] loss of consciousness |
| 839 |  | Closed fracture of vault of skull with other and unspecified intracranial hemorrhage, with concussion, unspecified |
| 84 |  | Closed fracture of vault of skull with intracranial injury of other and unspecified nature, unspecified state of consciousness |
| 841 |  | Closed fracture of vault of skull with intracranial injury of other and unspecified nature, with no loss of consciousness |
| 842 |  | Closed fracture of vault of skull with intracranial injury of other and unspecified nature, with brief [less than one hour] loss of consciousness |
| 843 |  | Closed fracture of vault of skull with intracranial injury of other and unspecified nature, with moderate [1-24 hours] loss of consciousness |
| 844 |  | Closed fracture of vault of skull with intracranial injury of other and unspecified nature, with prolonged [more than 24 hours] loss of consciousness and return to pre-existing conscious level |
| 845 |  | Closed fracture of vault of skull with intracranial injury of other and unspecified nature, with prolonged [more than 24 hours] loss of consciousness, without return to pre-existing conscious level |
| 846 |  | Closed fracture of vault of skull with intracranial injury of other and unspecified nature, with loss of consciousness of unspecified duration |
| 849 |  | Closed fracture of vault of skull with intracranial injury of other and unspecified nature, with concussion, unspecified |
| 85 |  | Open fracture of vault of skull without mention of intracranial injury, unspecified state of consciousness |
| 851 |  | Open fracture of vault of skull without mention of intracranial injury, with no loss of consciousness |
| 852 |  | Open fracture of vault of skull without mention of intracranial injury, with brief [less than one hour] loss of consciousness |
| 853 |  | Open fracture of vault of skull without mention of intracranial injury, with moderate [1-24 hours] loss of consciousness |
| 854 |  | Open fracture of vault of skull without mention of intracranial injury, with prolonged [more than 24 hours] loss of consciousness and return to pre-existing conscious level |
| 855 |  | Open fracture of vault of skull without mention of intracranial injury, with prolonged [more than 24 hours] loss of consciousness, without return to pre-existing conscious level |
| 856 |  | Open fracture of vault of skull without mention of intracranial injury, with loss of consciousness of unspecified duration |
| 859 |  | Open fracture of vault of skull without mention of intracranial injury, with concussion, unspecified |
| 86 |  | Open fracture of vault of skull with cerebral laceration and contusion, unspecified state of consciousness |
| 861 |  | Open fracture of vault of skull with cerebral laceration and contusion, with no loss of consciousness |
| 862 |  | Open fracture of vault of skull with cerebral laceration and contusion, with brief [less than one hour] loss of consciousness |
| 863 |  | Open fracture of vault of skull with cerebral laceration and contusion, with moderate [1-24 hours] loss of consciousness |
| 864 |  | Open fracture of vault of skull with cerebral laceration and contusion, with prolonged [more than 24 hours] loss of consciousness and return to pre-existing conscious level |
| 865 |  | Open fracture of vault of skull with cerebral laceration and contusion, with prolonged [more than 24 hours] loss of consciousness, without return to pre-existing conscious level |
| 866 |  | Open fracture of vault of skull with cerebral laceration and contusion, with loss of consciousness of unspecified duration |
| 869 |  | Open fracture of vault of skull with cerebral laceration and contusion, with concussion, unspecified |
| 87 |  | Open fracture of vault of skull with subarachnoid, subdural, and extradural hemorrhage, unspecified state of consciousness |
| 871 |  | Open fracture of vault of skull with subarachnoid, subdural, and extradural hemorrhage, with no loss of consciousness |
| 872 |  | Open fracture of vault of skull with subarachnoid, subdural, and extradural hemorrhage, with brief [less than one hour] loss of consciousness |
| 873 |  | Open fracture of vault of skull with subarachnoid, subdural, and extradural hemorrhage, with moderate [1-24 hours] loss of consciousness |
| 874 |  | Open fracture of vault of skull with subarachnoid, subdural, and extradural hemorrhage, with moderate [1-24 hours] loss of consciousness |
| 875 |  | Open fracture of vault of skull with subarachnoid, subdural, and extradural hemorrhage, with prolonged [more than 24 hours] loss of consciousness, without return to pre-existing conscious level |
| 876 |  | Open fracture of vault of skull with subarachnoid, subdural, and extradural hemorrhage, with loss of consciousness of unspecified duration |
| 879 |  | Open fracture of vault of skull with subarachnoid, subdural, and extradural hemorrhage, with concussion, unspecified |
| 88 |  | Open fracture of vault of skull with other and unspecified intracranial hemorrhage, unspecified state of consciousness |
| 881 |  | Open fracture of vault of skull with other and unspecified intracranial hemorrhage, with no loss of consciousness |
| 882 |  | Open fracture of vault of skull with other and unspecified intracranial hemorrhage, with brief [less than one hour] loss of |
| 883 |  | Open fracture of vault of skull with other and unspecified intracranial hemorrhage, with moderate [1-24 hours] loss of consciousness |
| 884 |  | Open fracture of vault of skull with other and unspecified intracranial hemorrhage, with prolonged [more than 24 hours] loss of consciousness and return to pre-existing conscious level |
| 885 |  | Open fracture of vault of skull with other and unspecified intracranial hemorrhage, with prolonged [more than 24 hours] loss of consciousness, without return to pre-existing conscious level |
| 886 |  | Open fracture of vault of skull with other and unspecified intracranial hemorrhage, with loss of consciousness of unspecified duration |
| 889 |  | Open fracture of vault of skull with other and unspecified intracranial hemorrhage, with concussion, unspecified |
| 89 |  | Open fracture of vault of skull with intracranial injury of other and unspecified nature, unspecified state of consciousness |
| 891 |  | Open fracture of vault of skull with intracranial injury of other and unspecified nature, with no loss of consciousness |
| 892 |  | Open fracture of vault of skull with intracranial injury of other and unspecified nature, with brief [less than one hour] loss of consciousness |
| 893 |  | Open fracture of vault of skull with intracranial injury of other and unspecified nature, with moderate [1-24 hours] loss of consciousness |
| 894 |  | Open fracture of vault of skull with intracranial injury of other and unspecified nature, with prolonged [more than 24 hours] loss of consciousness and return to pre-existing conscious level |
| 895 |  | Open fracture of vault of skull with intracranial injury of other and unspecified nature, with prolonged [more than 24 hours] loss of consciousness, without return to pre-existing conscious level |
| 896 |  | Open fracture of vault of skull with intracranial injury of other and unspecified nature, with loss of consciousness of unspecified duration |
| 899 |  | Open fracture of vault of skull with intracranial injury of other and unspecified nature, with concussion, unspecified |
| 81 |  | Closed fracture of base of skull without mention of intra cranial injury, unspecified state of consciousness |
| 811 |  | Closed fracture of base of skull without mention of intra cranial injury, with no loss of consciousness |
| 812 |  | Closed fracture of base of skull without mention of intra cranial injury, with brief [less than one hour] loss of consciousness |
| 813 |  | Closed fracture of base of skull without mention of intra cranial injury, with moderate [1-24 hours] loss of consciousness |
| 814 |  | Closed fracture of base of skull without mention of intra cranial injury, with prolonged [more than 24 hours] loss of consciousness and return to pre-existing conscious level |
| 815 |  | Closed fracture of base of skull without mention of intra cranial injury, with prolonged [more than 24 hours] loss of consciousness, without return to pre-existing conscious level |
| 816 |  | Closed fracture of base of skull without mention of intra cranial injury, with loss of consciousness of unspecified duration |
| 819 |  | Closed fracture of base of skull without mention of intra cranial injury, with concussion, unspecified |
| 811 |  | Closed fracture of base of skull with cerebral laceration and contusion, unspecified state of consciousness |
| 8111 |  | Closed fracture of base of skull with cerebral laceration and contusion, with no loss of consciousness |
| 8112 |  | Closed fracture of base of skull with cerebral laceration and contusion, with brief [less than one hour] loss of consciousness |
| 8113 |  | Closed fracture of base of skull with cerebral laceration and contusion, with moderate [1-24 hours] loss of consciousness |
| 8114 |  | Closed fracture of base of skull with cerebral laceration and contusion, with prolonged [more than 24 hours] loss of consciousness and return to pre-existing conscious level |
| 8115 |  | Closed fracture of base of skull with cerebral laceration and contusion, with prolonged [more than 24 hours] loss of consciousness, without return to pre-existing conscious level |
| 8116 |  | Closed fracture of base of skull with cerebral laceration and contusion, with loss of consciousness of unspecified duration |
| 8119 |  | Closed fracture of base of skull with cerebral laceration and contusion, with concussion, unspecified |
| 812 |  | Closed fracture of base of skull with subarachnoid, subdural, and extradural hemorrhage, unspecified state of consciousness |
| 8121 |  | Closed fracture of base of skull with subarachnoid, subdural, and extradural hemorrhage, with no loss of consciousness |
| 8122 |  | Closed fracture of base of skull with subarachnoid, subdural, and extradural hemorrhage, with brief [less than one hour] loss of consciousness |
| 8123 |  | Closed fracture of base of skull with subarachnoid, subdural, and extradural hemorrhage, with moderate [1-24 hours] loss of consciousness |
| 8124 |  | Closed fracture of base of skull with subarachnoid, subdural, and extradural hemorrhage, with prolonged [more than 24 hours] loss of consciousness and return to pre-existing conscious level |
| 83 |  | Other closed skull fracture without mention of intracranial injury, unspecified state of consciousness |
| 831 |  | Other closed skull fracture without mention of intracranial injury, with no loss of consciousness |
| 832 |  | Other closed skull fracture without mention of intracranial injury, with brief [less than one hour] loss of consciousness |
| 833 |  | Other closed skull fracture without mention of intracranial injury, with moderate [1-24 hours] loss of consciousness |
| 834 |  | Other closed skull fracture without mention of intracranial injury, with prolonged [more than 24 hours] loss of consciousness and return to pre-existing conscious level |
| 835 |  | Other closed skull fracture without mention of intracranial injury, with prolonged [more than 24 hours] loss of consciousness, without return to pre-existing conscious level |
| 836 |  | Other closed skull fracture without mention of intracranial injury, with loss of consciousness of unspecified duration |
| 839 |  | Other closed skull fracture without mention of intracranial injury, with concussion, unspecified |
| 831 |  | Other closed skull fracture with cerebral laceration and contusion, unspecified state of consciousness |
| 8311 |  | Other closed skull fracture with cerebral laceration and contusion, with no loss of consciousness |
| 8312 |  | Other closed skull fracture with cerebral laceration and contusion, with brief [less than one hour] loss of consciousness |
| 8313 |  | Other closed skull fracture with cerebral laceration and contusion, with moderate [1-24 hours] loss of consciousness |
| 8314 |  | Other closed skull fracture with cerebral laceration and contusion, with prolonged [more than 24 hours] loss of consciousness and return to pre-existing conscious level |
| 8315 |  | Other closed skull fracture with cerebral laceration and contusion, with prolonged [more than 24 hours] loss of consciousness, without return to pre-existing conscious level |
| 8316 |  | Other closed skull fracture with cerebral laceration and contusion, with loss of consciousness of unspecified duration |
| 8319 |  | Other closed skull fracture with cerebral laceration and contusion, with concussion, unspecified |
| 832 | 1 | Other closed skull fracture with subarachnoid, subdural, and extradural hemorrhage, unspecified state of consciousness |
| 8321 |  | Other closed skull fracture with subarachnoid, subdural, and extradural hemorrhage, with no loss of consciousness |
| 8322 |  | Other closed skull fracture with subarachnoid, subdural, and extradural hemorrhage, with brief [less than one hour] loss of consciousness |
| 8323 |  | Other closed skull fracture with subarachnoid, subdural, and extradural hemorrhage, with moderate [1-24 hours] loss of consciousness |
| 8324 |  | Other closed skull fracture with subarachnoid, subdural, and extradural hemorrhage, with prolonged [more than 24 hours] loss of consciousness and return to pre-existing conscious level |
| 8325 |  | Other closed skull fracture with subarachnoid, subdural, and extradural hemorrhage, with prolonged [more than 24 hours] loss of consciousness, without return to pre-existing conscious level |
| 8326 |  | Other closed skull fracture with subarachnoid, subdural, and extradural hemorrhage, with loss of consciousness of unspecified duration |
| 8329 |  | Other closed skull fracture with subarachnoid, subdural, and extradural hemorrhage, with concussion, unspecified |
| 833 |  | Other closed skull fracture with other and unspecified intracranial hemorrhage, unspecified state of unconsciousness |
| 8331 |  | Other closed skull fracture with other and unspecified intracranial hemorrhage, with no loss of consciousness |
| 8332 |  | Other closed skull fracture with other and unspecified intracranial hemorrhage, with brief [less than one hour] loss of consciousness |
| 8333 |  | Other closed skull fracture with other and unspecified intracranial hemorrhage, with moderate [1-24 hours] loss of consciousness |
| 8334 |  | Other closed skull fracture with other and unspecified intracranial hemorrhage, with prolonged [more than 24 hours] loss of consciousness and return to pre-existing conscious level |
| 8335 |  | Other closed skull fracture with other and unspecified intracranial hemorrhage, with prolonged [more than 24 hours] loss of consciousness, without return to pre-existing conscious level |
| 8336 |  | Other closed skull fracture with other and unspecified intracranial hemorrhage, with loss of consciousness of unspecified duration |
| 8339 |  | Other closed skull fracture with other and unspecified intracranial hemorrhage, with concussion, unspecified |
| 834 |  | Other closed skull fracture with intracranial injury of other and unspecified nature, unspecified state of consciousness |
| 8341 |  | Other closed skull fracture with intracranial injury of other and unspecified nature, with no loss of consciousness |
| 8342 |  | Other closed skull fracture with intracranial injury of other and unspecified nature, with brief [less than one hour] loss of consciousness |
| 8343 |  | Other closed skull fracture with intracranial injury of other and unspecified nature, with moderate [1-24 hours] loss of consciousness |
| 8344 |  | Other closed skull fracture with intracranial injury of other and unspecified nature, with prolonged [more than 24 hours] loss of consciousness and return to pre-existing conscious level |
| 8345 |  | Other closed skull fracture with intracranial injury of other and unspecified nature, with prolonged [more than 24 hours] loss of consciousness, without return to pre-existing conscious level |
| 853 |  | Concussion with prolonged loss of consciousness and return to pre-existing conscious level |
| 85193 |  | Other and unspecified cerebral laceration and contusion, with open intracranial wound, with moderate [1-24 hours] loss of consciousness |
| 85194 |  | Other and unspecified cerebral laceration and contusion, with open intracranial wound, with prolonged [more than 24 hours] loss of consciousness and return to pre-existing conscious level |
| 85195 |  | Other and unspecified cerebral laceration and contusion, with open intracranial wound, with prolonged [more than 24 hours] loss of consciousness without return to pre-existing conscious level |
| 85196 |  | Other and unspecified cerebral laceration and contusion, with open intracranial wound, with loss of consciousness of unspecified duration |
| 85199 |  | Other and unspecified cerebral laceration and contusion, with open intracranial wound, with concussion, unspecified |
| 852 |  | Subarachnoid hemorrhage following injury without mention of open intracranial wound, unspecified state of consciousness |
| 8521 |  | Subarachnoid hemorrhage following injury without mention of open intracranial wound, with no loss of consciousness |
| 85412 |  | Intracranial injury of other and unspecified nature with open intracranial wound, with brief [less than one hour] loss of consciousness |
| 85413 |  | Intracranial injury of other and unspecified nature with open intracranial wound, with moderate [1-24 hours] loss of consciousness |
| 85414 |  | Intracranial injury of other and unspecified nature with open intracranial wound, with prolonged [more than 24 hours] loss of consciousness and return to pre-existing conscious level |
| 85415 |  | Intracranial injury of other and unspecified nature with open intracranial wound, with prolonged [more than 24 hours] loss of consciousness without return to pre-existing conscious level |
| 85416 |  | Intracranial injury of other and unspecified nature with open intracranial wound, with loss of consciousness of unspecified duration |
| 85419 |  | Intracranial injury of other and unspecified nature with open intracranial wound, with concussion, unspecified |
| 8411 |  | Closed fractures involving skull or face with other bones, with cerebral laceration and contusion, with no loss of consciousness |
| 842 |  | Closed fractures involving skull or face with other bones with subarachnoid, subdural, and extradural hemorrhage, unspecified state of consciousness |
| 8421 |  | Closed fractures involving skull or face with other bones with subarachnoid, subdural, and extradural hemorrhage, with no loss of consciousness |
| 8422 |  | Closed fractures involving skull or face with other bones with subarachnoid, subdural, and extradural hemorrhage, with brief [less than one hour] loss of consciousness |
| 8423 |  | Closed fractures involving skull or face with other bones with subarachnoid, subdural, and extradural hemorrhage, with moderate [1-24 hours] loss of consciousness |
| 8424 |  | Closed fractures involving skull or face with other bones with subarachnoid, subdural, and extradural hemorrhage, with prolonged [more than 24 hours] loss of consciousness and return to pre-existing conscious level |
| 8425 |  | Closed fractures involving skull or face with other bones with subarachnoid, subdural, and extradural hemorrhage, with prolonged [more than 24 hours] loss of consciousness, without return to pre-existing conscious level |
| 8426 |  | Closed fractures involving skull or face with other bones with subarachnoid, subdural, and extradural hemorrhage, with loss of consciousness of unspecified duration |
| 8429 |  | Closed fractures involving skull or face with other bones with subarachnoid, subdural, and extradural hemorrhage, with concussion, unspecified |
| 843 |  | Closed fractures involving skull or face with other bones, with other and unspecified intracranial hemorrhage, unspecified state of consciousness |
| 8431 |  | Closed fractures involving skull or face with other bones, with other and unspecified intracranial hemorrhage, with no loss of consciousness |
| 8432 |  | Closed fractures involving skull or face with other bones, with other and unspecified intracranial hemorrhage, with brief [less than one hour] loss of consciousness |
| 8433 |  | Closed fractures involving skull or face with other bones, with other and unspecified intracranial hemorrhage, with moderate [1-24 hours] loss of consciousness |
| 8525 |  | Extradural hemorrhage following injury with open intracranial wound, unspecified state of consciousness |
| 85251 |  | Extradural hemorrhage following injury with open intracranial wound, with no loss of consciousness |
| 85252 |  | Extradural hemorrhage following injury with open intracranial wound, with brief [less than one hour] loss of consciousness |
| 85253 |  | Extradural hemorrhage following injury with open intracranial wound, with moderate [1-24 hours] loss of consciousness |
| 85254 |  | Extradural hemorrhage following injury with open intracranial wound, with prolonged [more than 24 hours] loss of consciousness and return to pre-existing conscious level |
| 85255 |  | Extradural hemorrhage following injury with open intracranial wound, with prolonged [more than 24 hours] loss of consciousness without return to pre-existing conscious level |
| 85256 |  | Extradural hemorrhage following injury with open intracranial wound, with loss of consciousness of unspecified duration |
| 85259 |  | Extradural hemorrhage following injury with open intracranial wound, with concussion, unspecified |
| 853 |  | Other and unspecified intracranial hemorrhage following injury without mention of open intracranial wound, unspecified state of consciousness |
| 8531 |  | Other and unspecified intracranial hemorrhage following injury without mention of open intracranial wound, with no loss of consciousness |
| 8532 |  | Other and unspecified intracranial hemorrhage following injury without mention of open intracranial wound, with brief [less than one hour] loss of consciousness |
| 8533 |  | Other and unspecified intracranial hemorrhage following injury without mention of open intracranial wound, with moderate [1-24 hours] loss of consciousness |
| 8534 |  | Other and unspecified intracranial hemorrhage following injury without mention of open intracranial wound, with prolonged [more than 24 hours] loss of consciousness and return to pre- existing conscious level |
| 8535 |  | Other and unspecified intracranial hemorrhage following injury without mention of open intracranial wound, with prolonged [more than 24 hours] loss of consciousness without return to pre-existing conscious level |
| 8536 |  | Other and unspecified intracranial hemorrhage following injury without mention of open intracranial wound, with loss of consciousness of unspecified duration |
| 8539 |  | Other and unspecified intracranial hemorrhage following injury without mention of open intracranial wound, with concussion, unspecified |
| 8531 |  | Other and unspecified intracranial hemorrhage following injury with open intracranial wound, unspecified state of consciousness |
| 85311 |  | Other and unspecified intracranial hemorrhage following injury with open intracranial wound, with no loss of consciousness |
| 85312 |  | Other and unspecified intracranial hemorrhage following injury with open intracranial wound, with brief [less than one hour] loss of consciousness |
| 85313 |  | Other and unspecified intracranial hemorrhage following injury with open intracranial wound, with moderate [1-24 hours] loss of consciousness |
| 85314 |  | Other and unspecified intracranial hemorrhage following injury with open intracranial wound, with prolonged [more than 24 hours] loss of consciousness and return to pre-existing conscious level |
| 85315 |  | Other and unspecified intracranial hemorrhage following injury with open intracranial wound, with prolonged [more than 24 hours] loss of consciousness without return to pre-existing conscious level |
| 85316 |  | Other and unspecified intracranial hemorrhage following injury with open intracranial wound, with loss of consciousness of unspecified duration |
| 85319 |  | Other and unspecified intracranial hemorrhage following injury with open intracranial wound, with concussion, unspecified |
| 854 |  | Intracranial injury of other and unspecified nature without mention of open intracranial wound, unspecified state of consciousness |
| 8541 |  | Intracranial injury of other and unspecified nature without mention of open intracranial wound, with no loss of consciousness |
| 8542 |  | Intracranial injury of other and unspecified nature without mention of open intracranial wound, with brief [less than one hour] loss of consciousness |
| 8543 |  | Intracranial injury of other and unspecified nature without mention of open intracranial wound, with moderate [1-24 hours] loss of consciousness |
| 8544 |  | Intracranial injury of other and unspecified nature without mention of open intracranial wound, with prolonged [more than 24 hours] loss of consciousness and return to pre-existing conscious level |
| 8545 |  | Intracranial injury of other and unspecified nature without mention of open intracranial wound, with prolonged [more than 24 hours] loss of consciousness without return to pre-existing conscious level |
| 8546 |  | Intracranial injury of other and unspecified nature without mention of open intracranial wound, with loss of consciousness of unspecified duration |
| 8549 |  | Intracranial injury of other and unspecified nature without mention of open intracranial wound, with concussion, unspecified |
| 8541 |  | Intracranial injury of other and unspecified nature with open intracranial wound, unspecified state of consciousness |
| 85411 |  | Intracranial injury of other and unspecified nature with open intracranial wound, with no loss of consciousness |
| 85412 |  | Intracranial injury of other and unspecified nature with open intracranial wound, with brief [less than one hour] loss of consciousness |
| 85413 |  | Intracranial injury of other and unspecified nature with open intracranial wound, with moderate [1-24 hours] loss of consciousness |
| 85414 |  | Intracranial injury of other and unspecified nature with open intracranial wound, with prolonged [more than 24 hours] loss of consciousness and return to pre-existing conscious level |
| 85415 |  | Intracranial injury of other and unspecified nature with open intracranial wound, with prolonged [more than 24 hours] loss of consciousness without return to pre-existing conscious level |
| 85416 |  | Intracranial injury of other and unspecified nature with open intracranial wound, with loss of consciousness of unspecified duration |
| 85419 |  | Intracranial injury of other and unspecified nature with open intracranial wound, with concussion, unspecified |
| 8434 |  | Closed fractures involving skull or face with other bones, with other and unspecified intracranial hemorrhage, with prolonged [more than 24 hours] loss of consciousness and return to pre- existing conscious level |
| 8435 |  | Closed fractures involving skull or face with other bones, with other and unspecified intracranial hemorrhage, with prolonged [more than 24 hours] loss of consciousness, without return to pre-existing conscious level |
| 8436 |  | Closed fractures involving skull or face with other bones, with other and unspecified intracranial hemorrhage, with loss of consciousness of unspecified duration |
| 8439 |  | Closed fractures involving skull or face with other bones, with other and unspecified intracranial hemorrhage, with concussion, unspecified |
| 844 |  | Closed fractures involving skull or face with other bones, with intracranial injury of other and unspecified nature, unspecified state of consciousness |
| 8441 |  | Closed fractures involving skull or face with other bones, with intracranial injury of other and unspecified nature, with no loss of consciousness |
| 8442 |  | Closed fractures involving skull or face with other bones, with intracranial injury of other and unspecified nature, with brief [less than one hour] loss of consciousness |
| 8443 |  | Closed fractures involving skull or face with other bones, with intracranial injury of other and unspecified nature, with moderate [1-24 hours] loss of consciousness |
| 8444 |  | Closed fractures involving skull or face with other bones, with intracranial injury of other and unspecified nature, with prolonged [more than 24 hours] loss of consciousness and return to pre-existing conscious level |
| 8445 |  | Closed fractures involving skull or face with other bones, with intracranial injury of other and unspecified nature, with prolonged [more than 24 hours] loss of consciousness, without return to pre-existing conscious level |
| 8446 |  | Closed fractures involving skull or face with other bones, with intracranial injury of other and unspecified nature, with loss of consciousness of unspecified duration |
| 8449 |  | Closed fractures involving skull or face with other bones, with intracranial injury of other and unspecified nature, with concussion, unspecified |
| 845 |  | Open fractures involving skull or face with other bones, without mention of intracranial injury, unspecified state of consciousness |
| 8451 |  | Open fractures involving skull or face with other bones, without mention of intracranial injury, with no loss of consciousness |
| 8452 |  | Open fractures involving skull or face with other bones, without mention of intracranial injury, with brief [less than one hour] loss of consciousness |
| 8453 |  | Open fractures involving skull or face with other bones, without mention of intracranial injury, with moderate [1-24 hours] loss of consciousness |
| 8454 |  | Open fractures involving skull or face with other bones, without mention of intracranial injury, with prolonged [more than 24 hours] loss of consciousness and return to pre-existing conscious level |
| 8455 |  | Open fractures involving skull or face with other bones, without mention of intracranial injury, with prolonged [more than 24 hours] loss of consciousness, without return to pre-existing conscious level |
| 8456 |  | Open fractures involving skull or face with other bones, without mention of intracranial injury, with loss of consciousness of unspecified duration |
| 8459 |  | Open fractures involving skull or face with other bones, without mention of intracranial injury, with concussion, unspecified |
| 846 |  | Open fractures involving skull or face with other bones, with cerebral laceration and contusion, unspecified state of consciousness |
| 8461 |  | Open fractures involving skull or face with other bones, with cerebral laceration and contusion, with no loss of consciousness |
| 8462 |  | Open fractures involving skull or face with other bones, with cerebral laceration and contusion, with brief [less than one hour] loss of consciousness |
| 8463 |  | Open fractures involving skull or face with other bones, with cerebral laceration and contusion, with moderate [1-24 hours] loss of consciousness |
| 8464 |  | Open fractures involving skull or face with other bones, with cerebral laceration and contusion, with prolonged [more than 24 hours] loss of consciousness and return to pre-existing conscious level |
| 8465 |  | Open fractures involving skull or face with other bones, with cerebral laceration and contusion, with prolonged [more than 24 hours] loss of consciousness, without return to pre-existing conscious level |
| 8466 |  | Open fractures involving skull or face with other bones, with cerebral laceration and contusion, with loss of consciousness of unspecified duration |
| 8469 |  | Open fractures involving skull or face with other bones, with cerebral laceration and contusion, with concussion, unspecified |
| 847 |  | Open fractures involving skull or face with other bones with subarachnoid, subdural, and extradural hemorrhage, unspecified state of consciousness |
| 8471 |  | Open fractures involving skull or face with other bones with subarachnoid, subdural, and extradural hemorrhage, with no loss of consciousness |
| 8472 |  | Open fractures involving skull or face with other bones with subarachnoid, subdural, and extradural hemorrhage, with brief [less than one hour] loss of consciousness |
| 8473 |  | Open fractures involving skull or face with other bones with subarachnoid, subdural, and extradural hemorrhage, with moderate [1-24 hours] loss of consciousness |
| 8513 |  | Cortex (cerebral) contusion without mention of open intracranial wound, with moderate [1-24 hours] loss of consciousness |
| 8514 |  | Cortex (cerebral) contusion without mention of open intracranial wound, with prolonged [more than 24 hours] loss of consciousness and return to pre-existing conscious level |
| 8515 |  | Cortex (cerebral) contusion without mention of open intracranial wound, with prolonged [more than 24 hours] loss of consciousness without return to pre-existing conscious level |
| 8516 |  | Cortex (cerebral) contusion without mention of open intracranial wound, with loss of consciousness of unspecified duration |
| 8346 |  | Other closed skull fracture with intracranial injury of other and unspecified nature, with loss of consciousness of unspecified duration |
| 8349 |  | Other closed skull fracture with intracranial injury of other and unspecified nature, with concussion, unspecified |
| 835 |  | Other open skull fracture without mention of injury, unspecified state of consciousness |
| 8351 |  | Other open skull fracture without mention of intracranial injury, with no loss of consciousness |
| 8352 |  | Other open skull fracture without mention of intracranial injury, with brief [less than one hour] loss of consciousness |
| 8353 |  | Other open skull fracture without mention of intracranial injury, with moderate [1-24 hours] loss of consciousness |
| 8354 |  | Other open skull fracture without mention of intracranial injury, with prolonged [more than 24 hours] loss of consciousness and return to pre-existing conscious level |
| 8355 |  | Other open skull fracture without mention of intracranial injury, with prolonged [more than 24 hours] loss of consciousness, without return to pre-existing conscious level |
| 8356 |  | Other open skull fracture without mention of intracranial injury, with loss of consciousness of unspecified duration |
| 8359 |  | Other open skull fracture without mention of intracranial injury, with concussion, unspecified |
| 836 |  | Other open skull fracture with cerebral laceration and contusion, unspecified state of consciousness |
| 8361 |  | Other open skull fracture with cerebral laceration and contusion, with no loss of consciousness |
| 8362 |  | Other open skull fracture with cerebral laceration and contusion, with brief [less than one hour] loss of consciousness |
| 8363 |  | Other open skull fracture with cerebral laceration and contusion, with moderate [1-24 hours] loss of consciousness |
| 8364 |  | Other open skull fracture with cerebral laceration and contusion, with prolonged [more than 24 hours] loss of consciousness and return to pre-existing conscious level |
| 8365 |  | Other open skull fracture with cerebral laceration and contusion, with prolonged [more than 24 hours] loss of consciousness, without return to pre-existing conscious level |
| 8366 |  | Other open skull fracture with cerebral laceration and contusion, with loss of consciousness of unspecified duration |
| 8369 |  | Other open skull fracture with cerebral laceration and contusion, with concussion, unspecified |
| 837 |  | Other open skull fracture with subarachnoid, subdural, and extradural hemorrhage, unspecified state of consciousness |
| 8371 |  | Other open skull fracture with subarachnoid, subdural, and extradural hemorrhage, with no loss of consciousness |
| 8372 |  | Other open skull fracture with subarachnoid, subdural, and extradural hemorrhage, with brief [less than one hour] loss of consciousness |
| 8373 |  | Other open skull fracture with subarachnoid, subdural, and extradural hemorrhage, with moderate [1-24 hours] loss of consciousness |
| 8374 |  | Other open skull fracture with subarachnoid, subdural, and extradural hemorrhage, with prolonged [more than 24 hours] loss of consciousness and return to pre-existing conscious level |
| 8375 |  | Other open skull fracture with subarachnoid, subdural, and extradural hemorrhage, with prolonged [more than 24 hours] loss of consciousness, without return to pre-existing conscious level |
| 8376 |  | Other open skull fracture with subarachnoid, subdural, and extradural hemorrhage, with loss of consciousness of unspecified duration |
| 8379 |  | Other open skull fracture with subarachnoid, subdural, and extradural hemorrhage, with concussion, unspecified |
| 838 |  | Other open skull fracture with other and unspecified intracranial hemorrhage, unspecified state of consciousness |
| 8381 |  | Other open skull fracture with other and unspecified intracranial hemorrhage, with no loss of consciousness |
| 8382 |  | Other open skull fracture with other and unspecified intracranial hemorrhage, with brief [less than one hour] loss of consciousness |
| 8383 |  | Other open skull fracture with other and unspecified intracranial hemorrhage, with moderate [1-24 hours] loss of consciousness |
| 8384 |  | Other open skull fracture with other and unspecified intracranial hemorrhage, with prolonged [more than 24 hours] loss of consciousness and return to pre-existing conscious level |
| 8385 |  | Other open skull fracture with other and unspecified intracranial hemorrhage, with prolonged [more than 24 hours] loss of consciousness, without return to pre-existing conscious level |
| 8386 |  | Other open skull fracture with other and unspecified intracranial hemorrhage, with loss of consciousness of unspecified duration |
| 8389 |  | Other open skull fracture with other and unspecified intracranial hemorrhage, with concussion, unspecified |
| 839 |  | Other open skull fracture with intracranial injury of other and unspecified nature, unspecified state of consciousness |
| 8391 |  | Other open skull fracture with intracranial injury of other and unspecified nature, with no loss of consciousness |
| 8392 |  | Other open skull fracture with intracranial injury of other and unspecified nature, with brief [less than one hour] loss of consciousness |
| 8393 |  | Other open skull fracture with intracranial injury of other and unspecified nature, with moderate [1-24 hours] loss of consciousness |
| 8394 |  | Other open skull fracture with intracranial injury of other and unspecified nature, with prolonged [more than 24 hours] loss of consciousness and return to pre-existing conscious level |
| 8395 |  | Other open skull fracture with intracranial injury of other and unspecified nature, with prolonged [more than 24 hours] loss of consciousness, without return to pre-existing conscious level |
| 8396 |  | Other open skull fracture with intracranial injury of other and unspecified nature, with loss of consciousness of unspecified duration |
| 8399 |  | Other open skull fracture with intracranial injury of other and unspecified nature, with concussion, unspecified |
| 84 |  | Closed fractures involving skull or face with other bones, without mention of intracranial injury, unspecified state of consciousness |
| 841 |  | Closed fractures involving skull or face with other bones, without mention of intracranial injury, with no loss of consciousness |
| 842 |  | Closed fractures involving skull or face with other bones, without mention of intracranial injury, with brief [less than one hour] loss of consciousness |
| 843 |  | Closed fractures involving skull or face with other bones, without mention of intracranial injury, with moderate [1-24 hours] loss of consciousness |
| 844 |  | Closed fractures involving skull or face with other bones, without mention or intracranial injury, with prolonged [more than 24 hours] loss of consciousness and return to pre-existing conscious level |
| 845 |  | Closed fractures involving skull of face with other bones, without mention of intracranial injury, with prolonged [more than 24 hours] loss of consciousness, without return to pre-existing conscious level |
| 846 |  | Closed fractures involving skull of face with other bones, without mention of intracranial injury, with loss of consciousness of unspecified duration |
| 849 |  | Closed fractures involving skull of face with other bones, without mention of intracranial injury, with concussion, unspecified |
| 841 |  | Closed fractures involving skull or face with other bones, with cerebral laceration and contusion, unspecified state of consciousness |
| 8474 |  | Open fractures involving skull or face with other bones with subarachnoid, subdural, and extradural hemorrhage, with prolonged [more than 24 hours] loss of consciousness and return to pre-existing conscious level |
| 8475 |  | Open fractures involving skull or face with other bones with subarachnoid, subdural, and extradural hemorrhage, with prolonged [more than 24 hours] loss of consciousness, without return to pre-existing conscious level |
| 8476 |  | Open fractures involving skull or face with other bones with subarachnoid, subdural, and extradural hemorrhage, with loss of consciousness of unspecified duration |
| 8479 |  | Open fractures involving skull or face with other bones with subarachnoid, subdural, and extradural hemorrhage, with concussion, unspecified |
| 848 |  | Open fractures involving skull or face with other bones, with other and unspecified intracranial hemorrhage, unspecified state of consciousness |
| 8481 |  | Open fractures involving skull or face with other bones, with other and unspecified intracranial hemorrhage, with no loss of consciousness |
| 8482 |  | Open fractures involving skull or face with other bones, with other and unspecified intracranial hemorrhage, with brief [less than one hour] loss of consciousness |
| 8483 |  | Open fractures involving skull or face with other bones, with other and unspecified intracranial hemorrhage, with moderate [1-24 hours] loss of consciousness |
| 8484 |  | Open fractures involving skull or face with other bones, with other and unspecified intracranial hemorrhage, with prolonged [more than 24 hours] loss of consciousness and return to pre-existing conscious level |
| 8485 |  | Open fractures involving skull or face with other bones, with other and unspecified intracranial hemorrhage, with prolonged [more than 24 hours] loss consciousness, without return to pre-existing conscious level |
| 8486 |  | Open fractures involving skull or face with other bones, with other and unspecified intracranial hemorrhage, with loss of consciousness of unspecified duration |
| 8489 |  | Open fractures involving skull or face with other bones, with other and unspecified intracranial hemorrhage, with concussion, unspecified |
| 849 |  | Open fractures involving skull or face with other bones, with intracranial injury of other and unspecified nature, unspecified state of consciousness |
| 8491 |  | Open fractures involving skull or face with other bones, with intracranial injury of other and unspecified nature, with no loss of consciousness |
| 8492 |  | Open fractures involving skull or face with other bones, with intracranial injury of other and unspecified nature, with brief [less than one hour] loss of consciousness |
| 8493 |  | Open fractures involving skull or face with other bones, with intracranial injury of other and unspecified nature, with moderate [1-24 hours] loss of consciousness |
| 8494 |  | Open fractures involving skull or face with other bones, with intracranial injury of other and unspecified nature, with prolonged [more than 24 hours] loss of consciousness and return to pre-existing conscious level |
| 8495 |  | Open fractures involving skull or face with other bones, with intracranial injury of other and unspecified nature, with prolonged [more than 24 hours] loss of consciousness without return to pre-existing conscious level |
| 8496 |  | Open fractures involving skull or face with other bones, with intracranial injury of other and unspecified nature, with loss of consciousness of unspecified duration |
| 8499 |  | Open fractures involving skull or face with other bones, with intracranial injury of other and unspecified nature, with concussion, unspecified |
| 8519 |  | Cortex (cerebral) contusion without mention of open intracranial wound, with concussion, unspecified |
| 8511 |  | Cortex (cerebral) contusion with open intracranial wound, unspecified state of consciousness |
| 85111 |  | Cortex (cerebral) contusion with open intracranial wound, with no loss of consciousness |
| 85112 |  | Cortex (cerebral) contusion with open intracranial wound, with brief [less than one hour] loss of consciousness |
| 85113 |  | Cortex (cerebral) contusion with open intracranial wound, with moderate [1-24 hours] loss of consciousness |
| 85114 |  | Cortex (cerebral) contusion with open intracranial wound, with prolonged [more than 24 hours] loss of consciousness and return to pre-existing conscious level |
| 85115 |  | Cortex (cerebral) contusion with open intracranial wound, with prolonged [more than 24 hours] loss of consciousness without return to pre-existing conscious level |
| 85116 |  | Cortex (cerebral) contusion with open intracranial wound, with loss of consciousness of unspecified duration |
| 85119 |  | Cortex (cerebral) contusion with open intracranial wound, with concussion, unspecified |
| 8512 |  | Cortex (cerebral) laceration without mention of open intracranial wound, unspecified state of consciousness |
| 85121 |  | Cortex (cerebral) laceration without mention of open intracranial wound, with no loss of consciousness |
| 85122 |  | Cortex (cerebral) laceration without mention of open intracranial wound, with brief [less than one hour] loss of consciousness |
| 85123 |  | Cortex (cerebral) laceration without mention of open intracranial wound, with moderate [1-24 hours] loss of consciousness |
| 85124 |  | Cortex (cerebral) laceration without mention of open intracranial wound, with prolonged [more than 24 hours] loss of consciousness and return to pre-existing conscious level |
| 85125 |  | Cortex (cerebral) laceration without mention of open intracranial wound, with prolonged [more than 24 hours] loss of consciousness |
| 85126 |  | Cortex (cerebral) laceration without mention of open intracranial wound, with loss of consciousness of unspecified duration |
| 85129 |  | Cortex (cerebral) laceration without mention of open intracranial wound, with concussion, unspecified |
| 8513 |  | Cortex (cerebral) laceration with open intracranial wound, unspecified state of consciousness |
| 85131 |  | Cortex (cerebral) laceration with open intracranial wound, with no loss of consciousness |
| 85132 |  | Cortex (cerebral) laceration with open intracranial wound, with brief [less than one hour] loss of consciousness |
| 85133 |  | Cortex (cerebral) laceration with open intracranial wound, with moderate [1-24 hours] loss of consciousness |
| 85134 |  | Cortex (cerebral) laceration with open intracranial wound, with prolonged [more than 24 hours] loss of consciousness and return to pre-existing conscious level |
| 85135 |  | Cortex (cerebral) laceration with open intracranial wound, with prolonged [more than 24 hours] loss of consciousness without return to pre-existing conscious level |
| 85136 |  | Cortex (cerebral) laceration with open intracranial wound, with loss of consciousness of unspecified duration |
| 85139 |  | Cortex (cerebral) laceration with open intracranial wound, with concussion, unspecified |
| 8514 |  | Cerebellar or brain stem contusion without mention of open intracranial wound, unspecified state of consciousness |
| 85141 |  | Cerebellar or brain stem contusion without mention of open intracranial wound, with no loss of consciousness |
| 85142 |  | Cerebellar or brain stem contusion without mention of open intracranial wound, with brief [less than one hour] loss of consciousness |
| 85143 |  | Cerebellar or brain stem contusion without mention of open intracranial wound, with moderate [1-24 hours] loss of consciousness |
| 85144 |  | Cerebellar or brain stem contusion without mention of open intracranial wound, with prolonged [more than 24 hours] loss consciousness and return to pre-existing conscious level |
| 85145 |  | Cerebellar or brain stem contusion without mention of open intracranial wound, with prolonged [more than 24 hours] loss of consciousness without return to pre-existing conscious level |
| 85146 |  | Cerebellar or brain stem contusion without mention of open intracranial wound, with loss of consciousness of unspecified duration |
| 85149 |  | Cerebellar or brain stem contusion without mention of open intracranial wound, with concussion, unspecified |
| 8515 |  | Cerebellar or brain stem contusion with open intracranial wound, unspecified state of consciousness |
| 85151 |  | Cerebellar or brain stem contusion with open intracranial wound, with no loss of consciousness |
| 85152 |  | Cerebellar or brain stem contusion with open intracranial wound, with brief [less than one hour] loss of consciousness |
| 85153 |  | Cerebellar or brain stem contusion with open intracranial wound, with moderate [1-24 hours] loss of consciousness |
