# Supplementary material for: Mechanical Learning for Prediction of Sepsis-Associated Encephalopathy
Source: Front Comput Neurosci. 2021 Nov 16;15:739265. doi: 10.3389/fncom.2021.739265 (PMC8636425; doi:10.3389/fncom.2021.739265)
Supplement: Supplementary Material 1 — Exclude patients with trauma of skull from the MIMIC-III database according to ICD9-codes. [file Data_Sheet_1.zip › Supplementary materials/Supplementary materials 10.DOCX]

| **Supplementary materials10** Infection site and ICD9code | | |  |  |  |  |  |  |  |  |  |
| --- | --- | --- | --- | --- | --- | --- | --- | --- | --- | --- | --- |
|  | ICD9code | Description |  |  |  |  |  |  |  |  |  |
| Intestinal infection |  |  |  |  |  |  |  |  |  |  |  |
|  | 845 | Intestinal infection due to Clostridium difficile | | | |  |  |  |  |  |  |
|  | 847 | Intestinal infection due to other gram-negative bacteria | | | | |  |  |  |  |  |
|  | 88 | Intestinal infection due to other organism, not elsewhere classified | | | | | |  |  |  |  |
|  | 90 | Infectious colitis, enteritis, and gastroenteritis | | | |  |  |  |  |  |  |
|  | 93 | Diarrhea of presumed infectious origin | | |  |  |  |  |  |  |  |
|  | 56081 | Intestinal or peritoneal adhesions with obstruction (postoperative) (postinfection) | | | | | | |  |  |  |
|  | 56982 | Ulceration of intestine | |  |  |  |  |  |  |  |  |
|  | 56983 | Perforation of intestine | |  |  |  |  |  |  |  |  |
|  | 800 | Intestinal infection due to E. coli, unspecified | |  |  |  |  |  |  |  |  |
|  | 801 | Intestinal infection due to enteropathogenic E. coli | |  |  |  |  |  |  |  |  |
|  | 802 | Intestinal infection due to enterotoxigenic E. coli | |  |  |  |  |  |  |  |  |
|  | 803 | Intestinal infection due to enteroinvasive E. coli | |  |  |  |  |  |  |  |  |
|  | 804 | Intestinal infection due to enterohemorrhagic E. coli | |  |  |  |  |  |  |  |  |
|  | 809 | Intestinal infection due to other intestinal E. coli infections | |  |  |  |  |  |  |  |  |
|  | 81 | Intestinal infection due to arizona group of paracolon bacilli | |  |  |  |  |  |  |  |  |
|  | 82 | Intestinal infection due to aerobacter aerogenes | |  |  |  |  |  |  |  |  |
|  | 83 | Intestinal infection due to proteus (mirabilis) (morganii) | |  |  |  |  |  |  |  |  |
|  | 841 | Intestinal infection due to staphylococcus | |  |  |  |  |  |  |  |  |
|  | 842 | Intestinal infection due to pseudomonas | |  |  |  |  |  |  |  |  |
|  | 843 | Intestinal infection due to campylobacter | |  |  |  |  |  |  |  |  |
|  | 844 | Intestinal infection due to yersinia enterocolitica | |  |  |  |  |  |  |  |  |
|  | 846 | Intestinal infection due to other anaerobes | |  |  |  |  |  |  |  |  |
|  | 849 | Intestinal infection due to other organisms | |  |  |  |  |  |  |  |  |
|  | 85 | Bacterial enteritis, unspecified | |  |  |  |  |  |  |  |  |
|  | 861 | Enteritis due to rotavirus | |  |  |  |  |  |  |  |  |
|  | 862 | Enteritis due to adenovirus | |  |  |  |  |  |  |  |  |
|  | 863 | Enteritis due to norwalk virus | |  |  |  |  |  |  |  |  |
|  | 864 | Enteritis due to other small round viruses [SRV's] | |  |  |  |  |  |  |  |  |
|  | 865 | Enteritis due to calicivirus | |  |  |  |  |  |  |  |  |
|  | 866 | Enteritis due to astrovirus | |  |  |  |  |  |  |  |  |
|  | 867 | Enteritis due to enterovirus nec | |  |  |  |  |  |  |  |  |
|  | 869 | Enteritis due to other viral enteritis | |  |  |  |  |  |  |  |  |
|  | 91 | Colitis, enteritis, and gastroenteritis of presumed infectious origin | |  |  |  |  |  |  |  |  |
|  | 92 | nfectious diarrhea | |  |  |  |  |  |  |  |  |
|  | 93 | Diarrhea of presumed infectious origin | |  |  |  |  |  |  |  |  |
|  | 5569 | Ulcerative colitis, unspecified | |  |  |  |  |  |  |  |  |
|  | 53100 | Acute gastric ulcer with hemorrhage, without mention of obstruction | |  |  |  |  |  |  |  |  |
|  | 53101 | Acute gastric ulcer with hemorrhage, with obstruction | |  |  |  |  |  |  |  |  |
|  | 53110 | Acute gastric ulcer with perforation, without mention of obstruction | |  |  |  |  |  |  |  |  |
|  | 53111 | Acute gastric ulcer with perforation, with obstruction | |  |  |  |  |  |  |  |  |
|  | 53120 | Acute gastric ulcer with hemorrhage and perforation, without mention of obstruction | |  |  |  |  |  |  |  |  |
|  | 53121 | Acute gastric ulcer with hemorrhage and perforation, with obstruction | |  |  |  |  |  |  |  |  |
|  | 53130 | Acute gastric ulcer without mention of hemorrhage or perforation, without mention of obstruction | |  |  |  |  |  |  |  |  |
|  | 53131 | Acute gastric ulcer without mention of hemorrhage or perforation, with obstruction | |  |  |  |  |  |  |  |  |
|  | 53161 | Acute salpingitis and oophoritis | |  |  |  |  |  |  |  |  |
|  | 53200 | Acute duodenal ulcer with hemorrhage, without mention of obstruction | |  |  |  |  |  |  |  |  |
|  | 53201 | Acute duodenal ulcer with hemorrhage, with obstruction | |  |  |  |  |  |  |  |  |
|  | 53210 | Acute duodenal ulcer with perforation, without mention of obstruction | |  |  |  |  |  |  |  |  |
|  | 53211 | Acute duodenal ulcer with perforation, with obstruction | |  |  |  |  |  |  |  |  |
|  | 53220 | Acute duodenal ulcer with hemorrhage and perforation, without mention of obstruction | |  |  |  |  |  |  |  |  |
|  | 53221 | Acute duodenal ulcer with hemorrhage and perforation, with obstruction | |  |  |  |  |  |  |  |  |
|  | 53231 | Acute duodenal ulcer without mention of hemorrhage or perforation, with obstruction | |  |  |  |  |  |  |  |  |
|  | 53290 | Duodenal ulcer, unspecified as acute or chronic, without hemorrhage or perforation, without mention of obstruction | |  |  |  |  |  |  |  |  |
|  | 53291 | Duodenal ulcer, unspecified as acute or chronic, without mention of hemorrhage or perforation, with obstruction | |  |  |  |  |  |  |  |  |
|  | 53300 | Acute peptic ulcer of unspecified site with hemorrhage, without mention of obstruction | |  |  |  |  |  |  |  |  |
|  | 53301 | Acute peptic ulcer of unspecified site with hemorrhage, with obstruction | |  |  |  |  |  |  |  |  |
|  | 53310 | Acute peptic ulcer of unspecified site with perforation, without mention of obstruction | |  |  |  |  |  |  |  |  |
|  | 53311 | Acute peptic ulcer of unspecified site with perforation, with obstruction | |  |  |  |  |  |  |  |  |
|  | 53320 | Acute peptic ulcer of unspecified site with hemorrhage and perforation, without mention of obstruction | |  |  |  |  |  |  |  |  |
|  | 53321 | Acute peptic ulcer of unspecified site with hemorrhage and perforation, with obstruction | |  |  |  |  |  |  |  |  |
|  | 53330 | Acute peptic ulcer of unspecified site without mention of hemorrhage and perforation, without mention of obstruction | |  |  |  |  |  |  |  |  |
|  | 53331 | Acute peptic ulcer of unspecified site without mention of hemorrhage and perforation, with obstruction | |  |  |  |  |  |  |  |  |
|  | 53400 | Acute gastrojejunal ulcer with hemorrhage, without mention of obstruction | |  |  |  |  |  |  |  |  |
|  | 53401 | Acute gastrojejunal ulcer, with hemorrhage, with obstruction | |  |  |  |  |  |  |  |  |
|  | 53410 | Acute gastrojejunal ulcer with perforation, without mention of obstruction | |  |  |  |  |  |  |  |  |
|  | 53411 | Acute gastrojejunal ulcer with perforation, with obstruction | |  |  |  |  |  |  |  |  |
|  | 53420 | Acute gastrojejunal ulcer with hemorrhage and perforation, without mention of obstruction | |  |  |  |  |  |  |  |  |
|  | 53421 | Acute gastrojejunal ulcer with hemorrhage and perforation, with obstruction | |  |  |  |  |  |  |  |  |
|  | 53430 | Acute gastrojejunal ulcer without mention of hemorrhage or perforation, without mention of obstruction | |  |  |  |  |  |  |  |  |
|  | 53431 | Acute gastrojejunal ulcer without mention of hemorrhage or perforation, with obstruction | |  |  |  |  |  |  |  |  |
|  | 53500 | Acute gastritis, without mention of hemorrhage | |  |  |  |  |  |  |  |  |
|  | 53501 | Acute gastritis, with hemorrhage | |  |  |  |  |  |  |  |  |
|  | 53560 | Duodenitis, without mention of hemorrhage | |  |  |  |  |  |  |  |  |
|  | 53561 | Duodenitis, with hemorrhage | |  |  |  |  |  |  |  |  |
|  | 53641 | Infection of gastrostomy | |  |  |  |  |  |  |  |  |
| Urinary infection |  |  |  |  |  |  |  |  |  |  |  |
|  | 5990 | Urinary tract infection | |  |  |  |  |  |  |  |  |
|  | 58089 | Acute glomerulonephritis with other specified pathological lesion in kidney | |  |  |  |  |  |  |  |  |
|  | 5809 | Acute glomerulonephritis with unspecified pathological lesion in kidney | |  |  |  |  |  |  |  |  |
|  | 5810 | Nephrotic syndrome with lesion of proliferative glomerulonephritis | |  |  |  |  |  |  |  |  |
|  | 5811 | Nephrotic syndrome with lesion of membranous glomerulonephritis | |  |  |  |  |  |  |  |  |
|  | 5812 | Nephrotic syndrome with lesion of membranoproliferative glomerulonephritis | |  |  |  |  |  |  |  |  |
|  | 5813 | Nephrotic syndrome with lesion of minimal change glomerulonephritis | |  |  |  |  |  |  |  |  |
|  | 5830 | Nephritis and nephropathy, not specified as acute or chronic, with lesion of proliferative glomerulonephritis | |  |  |  |  |  |  |  |  |
|  | 5831 | Nephritis and nephropathy, not specified as acute or chronic, with lesion of membranous glomerulonephritis | |  |  |  |  |  |  |  |  |
|  | 5832 | Nephritis and nephropathy, not specified as acute or chronic, with lesion of membranoproliferative glomerulonephritis | |  |  |  |  |  |  |  |  |
|  | 5834 | Nephritis and nephropathy, not specified as acute or chronic, with lesion of rapidly progressive glomerulonephritis | |  |  |  |  |  |  |  |  |
|  | 5836 | Nephritis and nephropathy, not specified as acute or chronic, with lesion of renal cortical necrosis | |  |  |  |  |  |  |  |  |
|  | 5837 | Nephritis and nephropathy, not specified as acute or chronic, with lesion of renal medullary necrosis | |  |  |  |  |  |  |  |  |
|  | 58381 | Nephritis and nephropathy, not specified as acute or chronic, in diseases classified elsewhere | |  |  |  |  |  |  |  |  |
|  | 58389 | Nephritis and nephropathy, not specified as acute or chronic, with other specified pathological lesion in kidney | |  |  |  |  |  |  |  |  |
|  | 5839 | Nephritis and nephropathy, not specified as acute or chronic, with unspecified pathological lesion in kidney | |  |  |  |  |  |  |  |  |
|  | 59010 | Acute pyelonephritis without lesion of renal medullary necrosis | |  |  |  |  |  |  |  |  |
|  | 59011 | Acute pyelonephritis with lesion of renal medullary necrosis | |  |  |  |  |  |  |  |  |
|  | 5902 | Renal and perinephric abscess | |  |  |  |  |  |  |  |  |
|  | 5903 | Pyeloureteritis cystica | |  |  |  |  |  |  |  |  |
|  | 59080 | Pyelonephritis, unspecified | |  |  |  |  |  |  |  |  |
|  | 59081 | Pyelitis or pyelonephritis in diseases classified elsewhere | |  |  |  |  |  |  |  |  |
|  | 5909 | Infection of kidney, unspecified | |  |  |  |  |  |  |  |  |
|  | 980 | Gonococcal infection (acute) of lower genitourinary tract | |  |  |  |  |  |  |  |  |
|  | 9810 | Gonococcal infection (acute) of upper genitourinary tract, site unspecified | |  |  |  |  |  |  |  |  |
|  | 9811 | Gonococcal cystitis (acute) | |  |  |  |  |  |  |  |  |
|  |  |  | |  |  |  |  |  |  |  |  |
| lung infection |  |  |  |  |  |  |  |  |  |  |  |
|  | 322 | Salmonella pneumonia |  |  |  |  |  |  |  |  |  |
|  | 1160 | Tuberculous pneumonia [any form], unspecified |  |  |  |  |  |  |  |  |  |
|  | 1161 | Tuberculous pneumonia [any form], bacteriological or histological examination not done |  |  |  |  |  |  |  |  |  |
|  | 1162 | Tuberculous pneumonia [any form], bacteriological or histological examination unknown (at present) | | |  |  |  |  |  |  |  |
|  | 1163 | Tuberculous pneumonia [any form], tubercle bacilli found (in sputum) by microscopy | | |  |  |  |  |  |  |  |
|  | 1164 | Tuberculous pneumonia [any form], tubercle bacilli not found (in sputum) by microscopy, but found by bacterial culture | | |  |  |  |  |  |  |  |
|  | 1165 | Tuberculous pneumonia [any form], tubercle bacilli not found by bacteriological examination, but tuberculosis confirmed histologically | | |  |  |  |  |  |  |  |
|  | 413 | Klebsiella pneumoniae | | |  |  |  |  |  |  |  |
|  | 551 | Postmeasles pneumonia | | |  |  |  |  |  |  |  |
|  | 382 | Pneumococcal septicemia [Streptococcus pneumoniae septicemia] | | |  |  |  |  |  |  |  |
|  | 11505 | Histoplasm caps pneumon | | |  |  |  |  |  |  |  |
|  | 11515 | Infection by Histoplasma duboisii, pneumonia | | |  |  |  |  |  |  |  |
|  | 11595 | Histoplasmosis, unspecified, pneumonia | | |  |  |  |  |  |  |  |
|  | 730 | Ornithosis with pneumonia | | |  |  |  |  |  |  |  |
|  | 48249 | Other Staphylococcus pneumonia | | |  |  |  |  |  |  |  |
|  | 48281 | Pneumonia due to anaerobes | | |  |  |  |  |  |  |  |
|  | 48282 | Pneumonia due to escherichia coli | | |  |  |  |  |  |  |  |
|  | 48283 | Pneumonia due to other gram-negative bacteria | | |  |  |  |  |  |  |  |
|  | 4800 | Pneumonia due to adenovirus | | |  |  |  |  |  |  |  |
|  | 4801 | Pneumonia due to respiratory syncytial virus | | |  |  |  |  |  |  |  |
|  | 4802 | Pneumonia due to parainfluenza virus | | |  |  |  |  |  |  |  |
|  | 4803 | Pneumonia due to SARS-associated coronavirus | | |  |  |  |  |  |  |  |
|  | 4808 | Pneumonia due to other virus not elsewhere classified | | |  |  |  |  |  |  |  |
|  | 4809 | Viral pneumonia, unspecified | | |  |  |  |  |  |  |  |
|  | 481 | Pneumococcal pneumonia [Streptococcus pneumoniae pneumonia] | | |  |  |  |  |  |  |  |
|  | 4820 | Pneumonia due to Klebsiella pneumoniae | | |  |  |  |  |  |  |  |
|  | 4821 | Pneumonia due to Pseudomonas | | |  |  |  |  |  |  |  |
|  | 4822 | Pneumonia due to Hemophilus influenzae [H. influenzae] | | |  |  |  |  |  |  |  |
|  | 48230 | Pneumonia due to Streptococcus, unspecified | | |  |  |  |  |  |  |  |
|  | 48231 | Pneumonia due to Streptococcus, group A | | |  |  |  |  |  |  |  |
|  | 48232 | Pneumonia due to Streptococcus, group B | | |  |  |  |  |  |  |  |
|  | 48239 | Pneumonia due to other Streptococcus | | |  |  |  |  |  |  |  |
|  | 48240 | Pneumonia due to Staphylococcus, unspecified | | |  |  |  |  |  |  |  |
|  | 48241 | Methicillin susceptible pneumonia due to Staphylococcus aureus | | |  |  |  |  |  |  |  |
|  | 48242 | Methicillin resistant pneumonia due to Staphylococcus aureus | | |  |  |  |  |  |  |  |
|  | 48284 | Pneumonia due to Legionnaires' disease | | |  |  |  |  |  |  |  |
|  | 48289 | Pneumonia due to other specified bacteria | | |  |  |  |  |  |  |  |
|  | 4829 | Bacterial pneumonia NOS Bacterial pneumonia, unspecified | | |  |  |  |  |  |  |  |
|  | 4830 | Pneumonia due to mycoplasma pneumoniae | | |  |  |  |  |  |  |  |
|  | 4831 | Pneumonia due to chlamydia | | |  |  |  |  |  |  |  |
|  | 4838 | Pneumonia due to other specified organism | | |  |  |  |  |  |  |  |
|  | 4841 | Pneumonia in cytomegalic inclusion disease | | |  |  |  |  |  |  |  |
|  | 4843 | Pneumonia in whoop cough | | |  |  |  |  |  |  |  |
|  | 4845 | Pneumonia in anthrax | | |  |  |  |  |  |  |  |
|  | 4846 | Pneum in aspergillosis | | |  |  |  |  |  |  |  |
|  | 4847 | Pneumonia in other systemic mycoses | | |  |  |  |  |  |  |  |
|  | 4848 | Pneumonia in other infectious diseases classified elsewhere | | |  |  |  |  |  |  |  |
|  | 485 | Bronchopneumonia, organism unspecified | | |  |  |  |  |  |  |  |
|  | 486 | Pneumonia, organism unspecified | | |  |  |  |  |  |  |  |
|  | 4870 | Influenza with pneumonia | | |  |  |  |  |  |  |  |
|  | 4871 | Influenza with other respiratory manifestations | | |  |  |  |  |  |  |  |
|  | 4878 | Influenza with other manifestations | | |  |  |  |  |  |  |  |
|  | 48801 | Influenza due to identified avian influenza virus with pneumonia | | |  |  |  |  |  |  |  |
|  | 48802 | Influenza due to identified avian influenza virus with other respiratory manifestations | | |  |  |  |  |  |  |  |
|  | 48809 | Influenza due to identified avian influenza virus with other manifestations | | |  |  |  |  |  |  |  |
|  | 48811 | Influenza due to identified 2009 H1N1 influenza virus with pneumonia | | |  |  |  |  |  |  |  |
|  | 48812 | Influenza due to identified 2009 H1N1 influenza virus with other respiratory manifestations | | |  |  |  |  |  |  |  |
|  | 48819 | Influenza due to identified 2009 H1N1 influenza virus with other manifestations | | |  |  |  |  |  |  |  |
|  | 48881 | Influenza due to identified novel influenza A virus with pneumonia | | |  |  |  |  |  |  |  |
|  | 48882 | Influenza due to identified novel influenza A virus with other respiratory manifestations | | |  |  |  |  |  |  |  |
|  | 48889 | Influenza due to identified novel influenza A virus with other manifestations | | |  |  |  |  |  |  |  |
|  | 51630 | Idiopathic interstitial pneumonia, not otherwise specified | | |  |  |  |  |  |  |  |
|  | 51635 | Idiopathic lymphoid interstitial pneumonia | | |  |  |  |  |  |  |  |
|  | 51636 | Cryptogenic organizing pneumonia | | |  |  |  |  |  |  |  |
|  | 51637 | Desquamative interstitial pneumonia | | |  |  |  |  |  |  |  |
|  | 5171 | Rheumatic pneumonia | | |  |  |  |  |  |  |  |
|  | 7700 | Congenital pneumonia | | |  |  |  |  |  |  |  |
|  | V066 | Need for prophylactic vaccination and inoculation against streptococcus pneumoniae [pneumococcus] and influenza | | |  |  |  |  |  |  |  |
|  | 99731 | Ventilator associated pneumonia | | |  |  |  |  |  |  |  |
|  | 99732 | Postprocedural aspiration pneumonia | | |  |  |  |  |  |  |  |
|  | V0382 | Other specified vaccinations against streptococcus pneumoniae [pneumococcus] | | |  |  |  |  |  |  |  |
|  | 1166 | Tuberculous pneumonia [any form], tubercle bacilli not found by bacteriological or histological examination, but tuberculosis confirmed by other methods [inoculation of animals] | | |  |  |  |  |  |  |  |
|  | 3453 | Grand mal status |  |  |  |  |  |  |  |  |  |
| Catheter-related |  |  |  |  |  |  |  |  |  |  |  |
|  |  |  |  |  |  |  |  |  |  |  |  |
|  | 99664 | Infection and inflammatory reaction due to indwelling urinary catheter |  |  |  |  |  |  |  |  |  |
|  | 99662 | Infection and inflammatory reaction due to other vascular device, implant, and graft |  |  |  |  |  |  |  |  |  |
|  | 99932 | Bloodstream infection due to central venous catheter |  |  |  |  |  |  |  |  |  |
|  | 99933 | Local infection due to central venous catheter |  |  |  |  |  |  |  |  |  |
|  | 99931 | Other and unspecified infection due to central venous catheter |  |  |  |  |  |  |  |  |  |
|  | 99668 | Infection and inflammatory reaction due to peritoneal dialysis catheter |  |  |  |  |  |  |  |  |  |
|  | 99731 | Ventilator associated pneumonia |  |  |  |  |  |  |  |  |  |
| Skin and soft tissue |  |  |  |  |  |  |  |  |  |  |  |
|  |  |  |  |  |  |  |  |  |  |  |  |
|  | 390 | Cutaneous actinomycotic infection |  |  |  |  |  |  |  |  |  |
|  | 6869 | Unspecified local infection of skin and subcutaneous tissue |  |  |  |  |  |  |  |  |  |
|  | 393 | Cervicofacial actinomycotic infection |  |  |  |  |  |  |  |  |  |
|  | 6868 | Other specified local infections of skin and subcutaneous tissue |  |  |  |  |  |  |  |  |  |
|  | 9161 | Abrasion or friction burn of hip, thigh, leg, and ankle, infected |  |  |  |  |  |  |  |  |  |
|  | 9159 | Other and unspecified superficial injury of fingers, infected |  |  |  |  |  |  |  |  |  |
|  | 9163 | Blister of hip, thigh, leg, and ankle, infected |  |  |  |  |  |  |  |  |  |
|  | 9165 | Insect bite, nonvenomous of hip, thigh, leg, and ankle, infected |  |  |  |  |  |  |  |  |  |
|  | 9167 | Superficial foreign body (splinter) of hip, thigh, leg, and ankle, without major open wound, infected |  |  |  |  |  |  |  |  |  |
|  | 9169 | Other and unspecified superficial injury of hip, thigh, leg, and ankle, infected |  |  |  |  |  |  |  |  |  |
|  | 9145 | Insect bite, nonvenomous, of hand(s) except finger(s) alone, infected |  |  |  |  |  |  |  |  |  |
|  | 9147 | Superficial foreign body (splinter) of hand(s) except finger(s) alone, without major open wound, infected |  |  |  |  |  |  |  |  |  |
|  | 9149 | Other and unspecified superficial injury of hand(s) except finger(s) alone, infected |  |  |  |  |  |  |  |  |  |
|  | 9151 | Abrasion or friction burn of finger(s), infected |  |  |  |  |  |  |  |  |  |
|  | 9153 | Blister of finger(s), infected |  |  |  |  |  |  |  |  |  |
|  | 9157 | Superficial foreign body (splinter) of finger(s), without major open wound, infected |  |  |  |  |  |  |  |  |  |
|  | 9197 | Superficial foreign body (splinter) of other, multiple, and unspecified sites, without major open wound, infected |  |  |  |  |  |  |  |  |  |
|  | 9199 | Other and unspecified superficial injury of other, multiple, and unspecified sites, infected |  |  |  |  |  |  |  |  |  |
|  | 9105 | Insect bite, nonvenomous of face, neck, and scalp except eye, infected |  |  |  |  |  |  |  |  |  |
|  | 9127 | Superficial foreign body (splinter) of shoulder and upper arm, without major open wound, infected |  |  |  |  |  |  |  |  |  |
|  | 9129 | Other and unspecified superficial injury of shoulder and upper arm, infected |  |  |  |  |  |  |  |  |  |
|  | 9131 | Abrasion or friction burn of elbow, forearm, and wrist, infected |  |  |  |  |  |  |  |  |  |
|  | 9133 | Blister of elbow, forearm, and wrist, infected |  |  |  |  |  |  |  |  |  |
|  | 9135 | Insect bite, nonvenomous, of elbow, forearm, and wrist, infected |  |  |  |  |  |  |  |  |  |
|  | 9137 | Superficial foreign body (splinter) of elbow, forearm, and wrist, without major open wound, infected |  |  |  |  |  |  |  |  |  |
|  | 9107 | Superficial foreign body (splinter) of face, neck, and scalp except eye, without major open wound, infected |  |  |  |  |  |  |  |  |  |
|  | 9109 | Other and unspecified superficial injury of face, neck, and scalp, infected |  |  |  |  |  |  |  |  |  |
|  | 9111 | Abrasion or friction burn of trunk, infected |  |  |  |  |  |  |  |  |  |
|  | 9113 | Blister of trunk, infected |  |  |  |  |  |  |  |  |  |
|  | 9115 | Insect bite, nonvenomous of trunk, infected |  |  |  |  |  |  |  |  |  |
|  | 9117 | Superficial foreign body (splinter) of trunk, without major open wound, infected |  |  |  |  |  |  |  |  |  |
|  | 9119 | Other and unspecified superficial injury of trunk, infected |  |  |  |  |  |  |  |  |  |
|  | 9121 | Abrasion or friction burn of shoulder and upper arm, infected |  |  |  |  |  |  |  |  |  |
|  | 9123 | Blister of shoulder and upper arm, infected |  |  |  |  |  |  |  |  |  |
|  | 9125 | Insect bite, nonvenomous of shoulder and upper arm, infected |  |  |  |  |  |  |  |  |  |
|  | 9139 | Other and unspecified superficial injury of elbow, forearm, and wrist, infected |  |  |  |  |  |  |  |  |  |
|  | 9141 | Abrasion or friction burn of hand(s) except finger(s) alone, infected |  |  |  |  |  |  |  |  |  |
|  | 9171 | Abrasion or friction burn of foot and toe(s), infected |  |  |  |  |  |  |  |  |  |
|  | 9173 | Blister of foot and toe(s), infected |  |  |  |  |  |  |  |  |  |
|  | 9175 | Insect bite, nonvenomous, of foot and toe(s), infected |  |  |  |  |  |  |  |  |  |
|  | 9177 | Superficial foreign body (splinter) of foot and toe(s), without major open wound, infected |  |  |  |  |  |  |  |  |  |
|  | 9179 | Other and unspecified superficial injury of foot and toes, infected |  |  |  |  |  |  |  |  |  |
|  | 9101 | brasion or friction burn of face, neck, and scalp except eye, infected |  |  |  |  |  |  |  |  |  |
|  | 9103 | Blister of face, neck, and scalp except eye, infected |  |  |  |  |  |  |  |  |  |
|  | 1118 | Other specified dermatomycoses |  |  |  |  |  |  |  |  |  |
|  | 1119 | Dermatomycosis, unspecified |  |  |  |  |  |  |  |  |  |
|  | 1121 | Candidiasis of vulva and vagina |  |  |  |  |  |  |  |  |  |
|  | 56731 | Psoas muscle abscess |  |  |  |  |  |  |  |  |  |
|  | 5283 | Cellulitis and abscess of oral soft tissues |  |  |  |  |  |  |  |  |  |
|  | 6943 | Impetigo herpetiformis |  |  |  |  |  |  |  |  |  |
|  | 6940 | Dermatitis herpetiformis |  |  |  |  |  |  |  |  |  |
|  | 6948 | Other specified bullous dermatoses |  |  |  |  |  |  |  |  |  |
|  | 6800 | Carbuncle and furuncle of face |  |  |  |  |  |  |  |  |  |
|  | 6801 | Carbuncle and furuncle of neck |  |  |  |  |  |  |  |  |  |
|  | 6803 | Carbuncle and furuncle of upper arm and forearm |  |  |  |  |  |  |  |  |  |
|  | 6804 | Carbuncle and furuncle of hand |  |  |  |  |  |  |  |  |  |
|  | 6805 | Carbuncle and furuncle of buttock |  |  |  |  |  |  |  |  |  |
|  | 6806 | Carbuncle and furuncle of leg, except foot |  |  |  |  |  |  |  |  |  |
|  | 6807 | Carbuncle and furuncle of foot |  |  |  |  |  |  |  |  |  |
|  | 68100 | Cellulitis and abscess of finger, unspecified |  |  |  |  |  |  |  |  |  |
|  | 6820 | Cellulitis and abscess of face |  |  |  |  |  |  |  |  |  |
|  | 6821 | Cellulitis and abscess of neck |  |  |  |  |  |  |  |  |  |
|  | 6822 | Cellulitis and abscess of trunk |  |  |  |  |  |  |  |  |  |
|  | 6823 | Cellulitis and abscess of upper arm and forearm |  |  |  |  |  |  |  |  |  |
|  | 6824 | Cellulitis and abscess of hand, except fingers and thumb |  |  |  |  |  |  |  |  |  |
|  | 6825 | Cellulitis and abscess of buttock |  |  |  |  |  |  |  |  |  |
|  | 6826 | Cellulitis and abscess of leg, except foot |  |  |  |  |  |  |  |  |  |
|  | 6827 | Cellulitis and abscess of foot, except toes |  |  |  |  |  |  |  |  |  |
|  | 6850 | Pilonidal cyst with abscess |  |  |  |  |  |  |  |  |  |
|  | 68600 | Pyoderma, unspecified |  |  |  |  |  |  |  |  |  |
|  | 70710 | Ulcer of lower limb, unspecified |  |  |  |  |  |  |  |  |  |
|  | 70711 | Ulcer of thigh |  |  |  |  |  |  |  |  |  |
|  | 70712 | Ulcer of calf |  |  |  |  |  |  |  |  |  |
|  | 70713 | Ulcer of ankle |  |  |  |  |  |  |  |  |  |
|  | 70714 | Ulcer of heel and midfoot |  |  |  |  |  |  |  |  |  |
|  | 70715 | Ulcer of other part of foot |  |  |  |  |  |  |  |  |  |
|  | 70719 | Ulcer of other part of lower limb |  |  |  |  |  |  |  |  |  |
|  | 70720 | Pressure ulcer, unspecified stage |  |  |  |  |  |  |  |  |  |
| Abdominal cavity |  |  |  |  |  |  |  |  |  |  |  |
|  | 1220 | Isolated tracheal or bronchial tuberculosis, unspecified |  |  |  |  |  |  |  |  |  |
|  | 1225 | Isolated tracheal or bronchial tuberculosis, tubercle bacilli not found by bacteriological examination, but tuberculosis confirmed histologically |  |  |  |  |  |  |  |  |  |
|  | 56721 | Peritonitis (acute) generalized |  |  |  |  |  |  |  |  |  |
|  | 56722 | Peritoneal abscess |  |  |  |  |  |  |  |  |  |
|  | 56723 | Spontaneous bacterial peritonitis |  |  |  |  |  |  |  |  |  |
|  | 56729 | Other suppurative peritonitis |  |  |  |  |  |  |  |  |  |
|  | 56738 | Other retroperitoneal abscess |  |  |  |  |  |  |  |  |  |
|  | 56739 | Other retroperitoneal infections |  |  |  |  |  |  |  |  |  |
|  | 5680 | Peritoneal adhesions (postoperative) (postinfection) |  |  |  |  |  |  |  |  |  |
|  | 56961 | Infection of colostomy or enterostomy |  |  |  |  |  |  |  |  |  |
|  | 56982 | Ulceration of intestine |  |  |  |  |  |  |  |  |  |
|  | 56983 | Perforation of intestine |  |  |  |  |  |  |  |  |  |
|  | 57400 | Calculus of gallbladder with acute cholecystitis, without mention of obstruction |  |  |  |  |  |  |  |  |  |
|  | 57401 | Calculus of gallbladder with acute cholecystitis, with obstruction |  |  |  |  |  |  |  |  |  |
|  | 57410 | Calculus of gallbladder with other cholecystitis, without mention of obstruction |  |  |  |  |  |  |  |  |  |
|  | 57411 | Calculus of gallbladder with other cholecystitis, with obstruction |  |  |  |  |  |  |  |  |  |
|  | 57430 | Calculus of bile duct with acute cholecystitis, without mention of obstruction |  |  |  |  |  |  |  |  |  |
|  | 57431 | Calculus of bile duct with acute cholecystitis, with obstruction |  |  |  |  |  |  |  |  |  |
|  | 57440 | Calculus of bile duct with other cholecystitis, without mention of obstruction |  |  |  |  |  |  |  |  |  |
|  | 57441 | Calculus of bile duct with other cholecystitis, with obstruction |  |  |  |  |  |  |  |  |  |
|  | 57460 | Calculus of gallbladder and bile duct with acute cholecystitis, without mention of obstruction |  |  |  |  |  |  |  |  |  |
|  | 57461 | Calculus of gallbladder and bile duct with acute cholecystitis, with obstruction |  |  |  |  |  |  |  |  |  |
|  | 57470 | Calculus of gallbladder and bile duct with other cholecystitis, without mention of obstruction |  |  |  |  |  |  |  |  |  |
|  | 57471 | Calculus of gallbladder and bile duct with other cholecystitis, with obstruction |  |  |  |  |  |  |  |  |  |
|  | 57480 | Calculus of gallbladder and bile duct with acute and chronic cholecystitis, without mention of obstruction |  |  |  |  |  |  |  |  |  |
|  | 57481 | Calculus of gallbladder and bile duct with acute and chronic cholecystitis, with obstruction |  |  |  |  |  |  |  |  |  |
|  | 5750 | Acute cholecystitis |  |  |  |  |  |  |  |  |  |
|  | 57510 | Cholecystitis, unspecified |  |  |  |  |  |  |  |  |  |
|  | 57512 | Acute and chronic cholecystitis |  |  |  |  |  |  |  |  |  |
|  | 5754 | Perforation of gallbladder |  |  |  |  |  |  |  |  |  |
|  | 5761 | Cholangitis |  |  |  |  |  |  |  |  |  |
|  | 5763 | Perforation of bile duct |  |  |  |  |  |  |  |  |  |
|  | 5764 | Fistula of bile duct |  |  |  |  |  |  |  |  |  |
|  | 5770 | Acute pancreatitis |  |  |  |  |  |  |  |  |  |
|  | 5400 | Acute appendicitis with generalized peritonitis |  |  |  |  |  |  |  |  |  |
|  | 5401 | Acute appendicitis with peritoneal abscess |  |  |  |  |  |  |  |  |  |
|  | 5409 | Acute appendicitis without mention of peritonitis |  |  |  |  |  |  |  |  |  |
|  | 541 | Appendicitis, unqualified |  |  |  |  |  |  |  |  |  |
|  | 542 | Other appendicitis |  |  |  |  |  |  |  |  |  |
|  | 55000 | Inguinal hernia, with gangrene, unilateral or unspecified (not specified as recurrent) |  |  |  |  |  |  |  |  |  |
|  | 55001 | Inguinal hernia, with gangrene, unilateral or unspecified, recurrent |  |  |  |  |  |  |  |  |  |
|  | 55002 | Inguinal hernia, with gangrene, bilateral (not specified as recurrent) |  |  |  |  |  |  |  |  |  |
|  | 55003 | Inguinal hernia, with gangrene, bilateral, recurrent |  |  |  |  |  |  |  |  |  |
|  | 55100 | Femoral hernia with gangrene, unilateral or unspecified (not specified as recurrent) |  |  |  |  |  |  |  |  |  |
|  | 55101 | Femoral hernia with gangrene, unilateral or unspecified, recurrent |  |  |  |  |  |  |  |  |  |
|  | 55102 | Femoral hernia with gangrene, bilateral (not specified as recurrent) |  |  |  |  |  |  |  |  |  |
|  | 55103 | Femoral hernia with gangrene, bilateral, recurrent |  |  |  |  |  |  |  |  |  |
|  | 5511 | Umbilical hernia with gangrene |  |  |  |  |  |  |  |  |  |
|  | 55120 | Ventral hernia, unspecified, with gangrene |  |  |  |  |  |  |  |  |  |
|  | 55121 | Incisional ventral hernia, with gangrene |  |  |  |  |  |  |  |  |  |
|  | 55129 | Other ventral hernia with gangrene |  |  |  |  |  |  |  |  |  |
|  | 5513 | Diaphragmatic hernia with gangrene |  |  |  |  |  |  |  |  |  |
|  | 5518 | Hernia of other specified sites, with gangrene |  |  |  |  |  |  |  |  |  |
|  | 5519 | Hernia of unspecified site, with gangrene |  |  |  |  |  |  |  |  |  |
|  | 6146 | Pelvic peritoneal adhesions, female (postoperative) (postinfection) |  |  |  |  |  |  |  |  |  |
|  | 6147 | Other chronic pelvic peritonitis, female |  |  |  |  |  |  |  |  |  |
|  |  |  |  |  |  |  |  |  |  |  |  |
