# Supplementary material for: Mechanical Learning for Prediction of Sepsis-Associated Encephalopathy
Source: Front Comput Neurosci. 2021 Nov 16;15:739265. doi: 10.3389/fncom.2021.739265 (PMC8636425; doi:10.3389/fncom.2021.739265)
Supplement: Supplementary Material 1 — Exclude patients with trauma of skull from the MIMIC-III database according to ICD9-codes. [file Data_Sheet_1.zip › Supplementary materials/Supplementary materials 12.DOCX]

Data Profiling Report

- Basic Statistics
- Raw Counts
- Percentages
- Data Structure
- Missing Data Profile
- Univariate Distribution
- Histogram
- Bar Chart (by frequency)
- QQ Plot
- Correlation Analysis
- Principal Component Analysis

Basic Statistics

| Raw Counts | |
| --- | --- |
| **Name** | **Value** |
| Rows | 2,535 |
| Columns | 33 |
| Discrete columns | 2 |
| Continuous columns | 31 |
| All missing columns | 0 |
| Missing observations | 473 |
| Complete Rows | 2,148 |
| Total observations | 83,655 |
| Memory allocation | 483.6 Kb |

Percentages

Memory Usage: 483.6 Kb


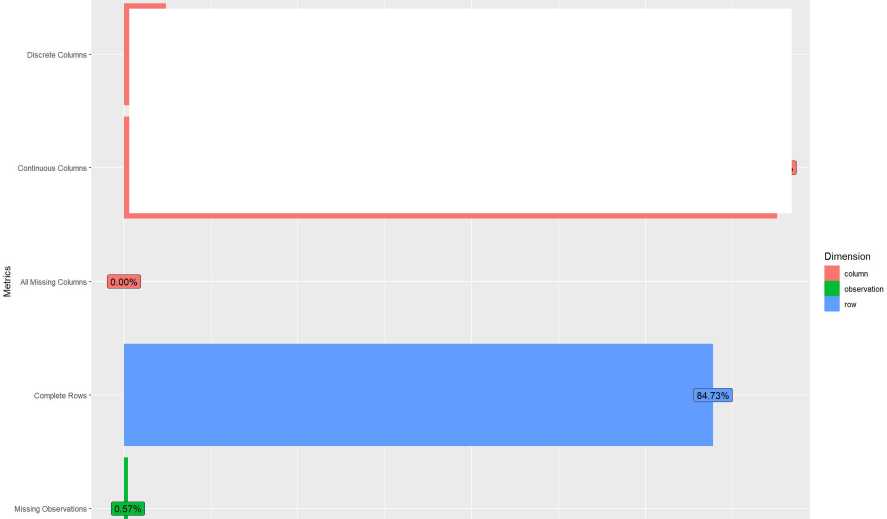

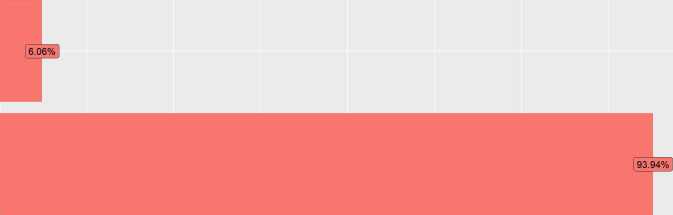


25%

75%

50%

Value

Data Structure

°subject id (int)

-—oMingcsfint)

oGrom) (mt)

°ICU.fos (num)

oGender (Factor w/ 2 levels ^o^qsofa (int)

°SOFA(int)

oSAPSII (int)

°Heartrate (int)

°Sysbp (num)

°Diasbp (int)

°Resprate (int)

°Teinpc (num)

°PH (num)

-^WBC (niun)

root (Classes 'data.table' and 'data.frame^1^: 2535 obs. of 33 variables:)^0^-^

°Monocytes (num)

°Neutrophils (num)

°Lymphocytes (num)

°Eosinophils (num)

°Blood.urea.nitrogen (int)

° Aspartate. aminotransferase (int)

°Alanine. aminotransferase (int)

~~ Albumin (num)

oPartial.thromboplastin.time (num) °Potassium (num)

'Sodium (im)

°Lac (nuni) ^o^Hemoglobin (num) oPlatel畠(int) °Glucose (int) °Age (num) °gender (Factor w/ 2 levels

Missing Data Profile

Eosinophils

Lymphocytes

Neutrophils

Aspanate.aminotransferase

Glucose

Blood.urea.nitrogen

[o%]区II・・・・・・・・・・・・・EI[O%]II

Potassium

Monocytes

Partial.thromboplastm.time

Hemoglobin

Univariate Distribution

Histogram


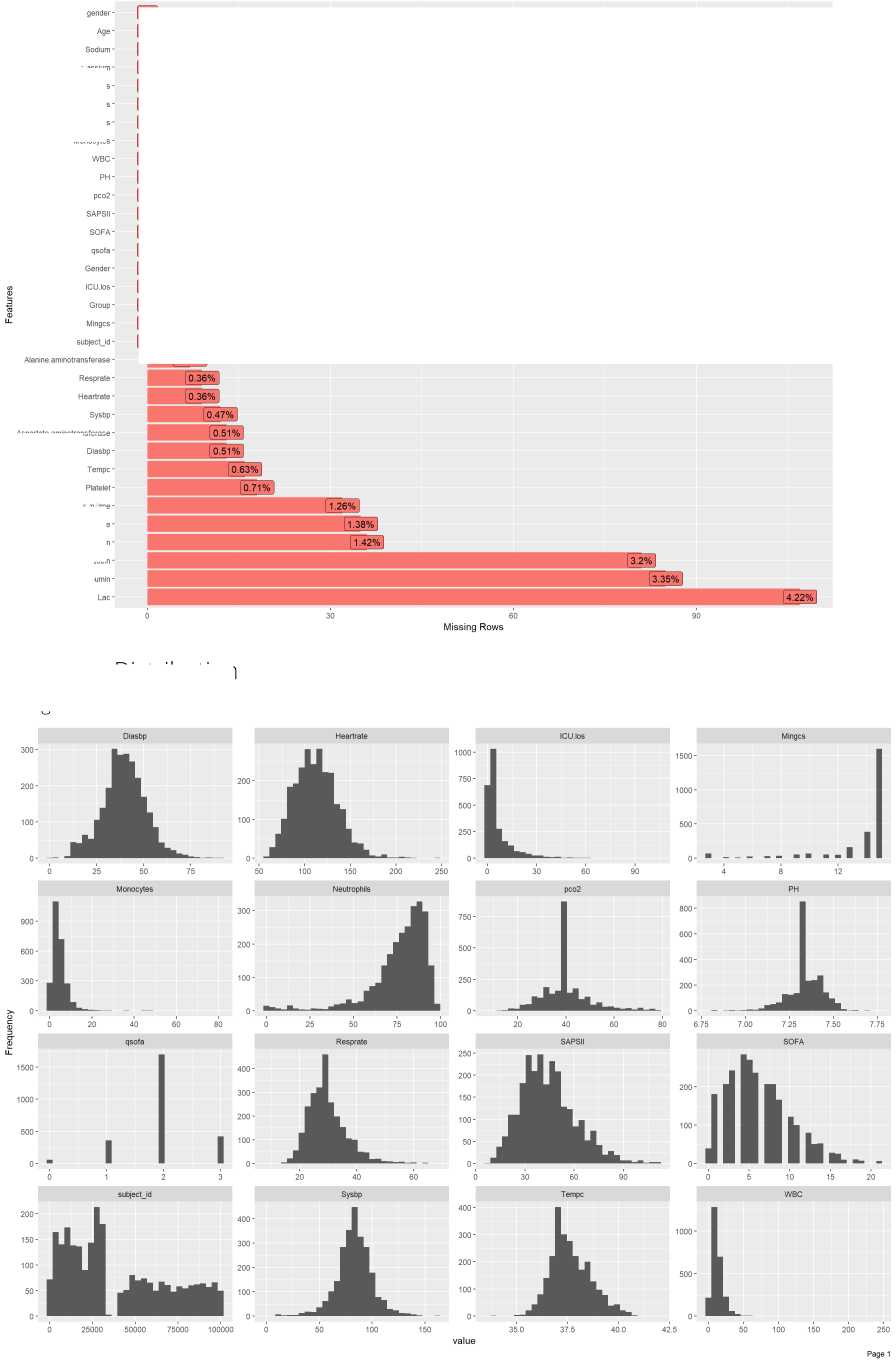

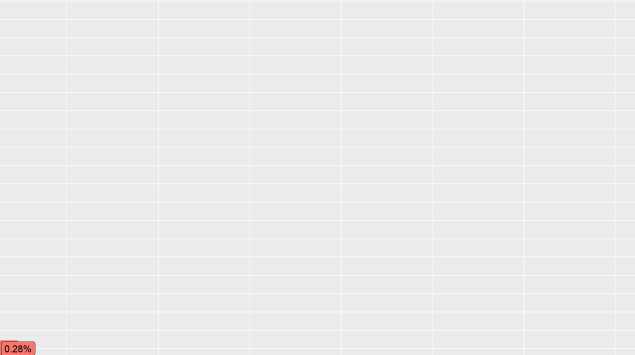

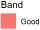


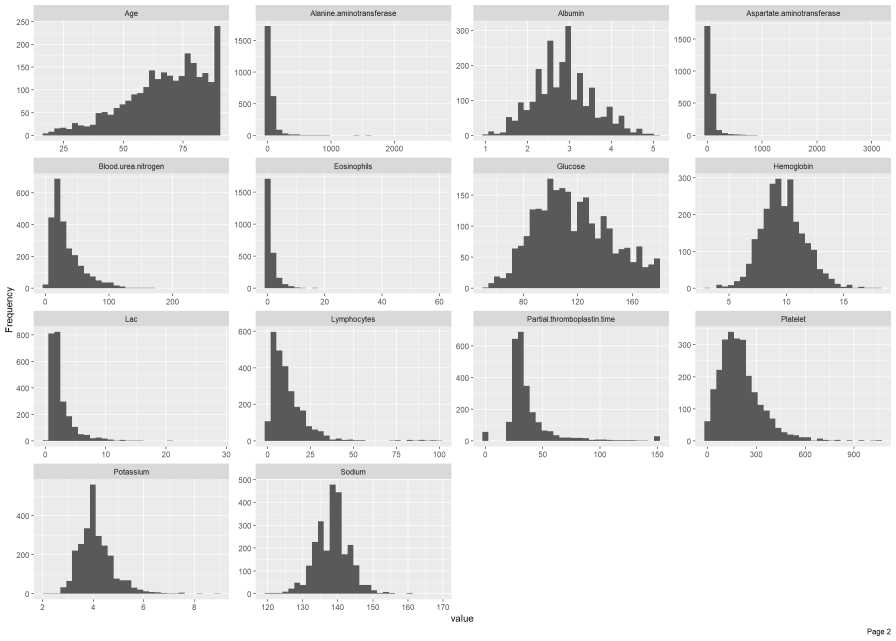


Bar Chart (by frequency)


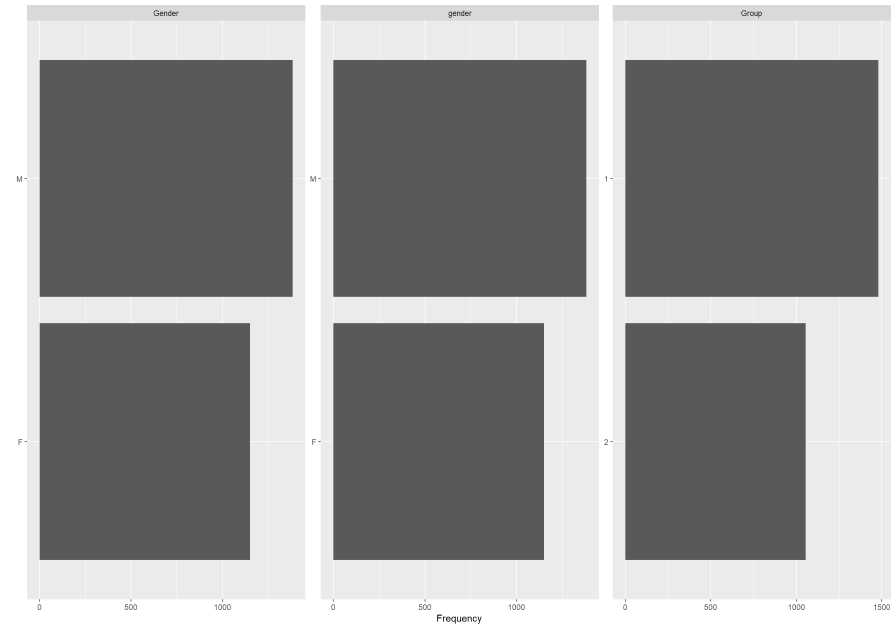


QQ Plot


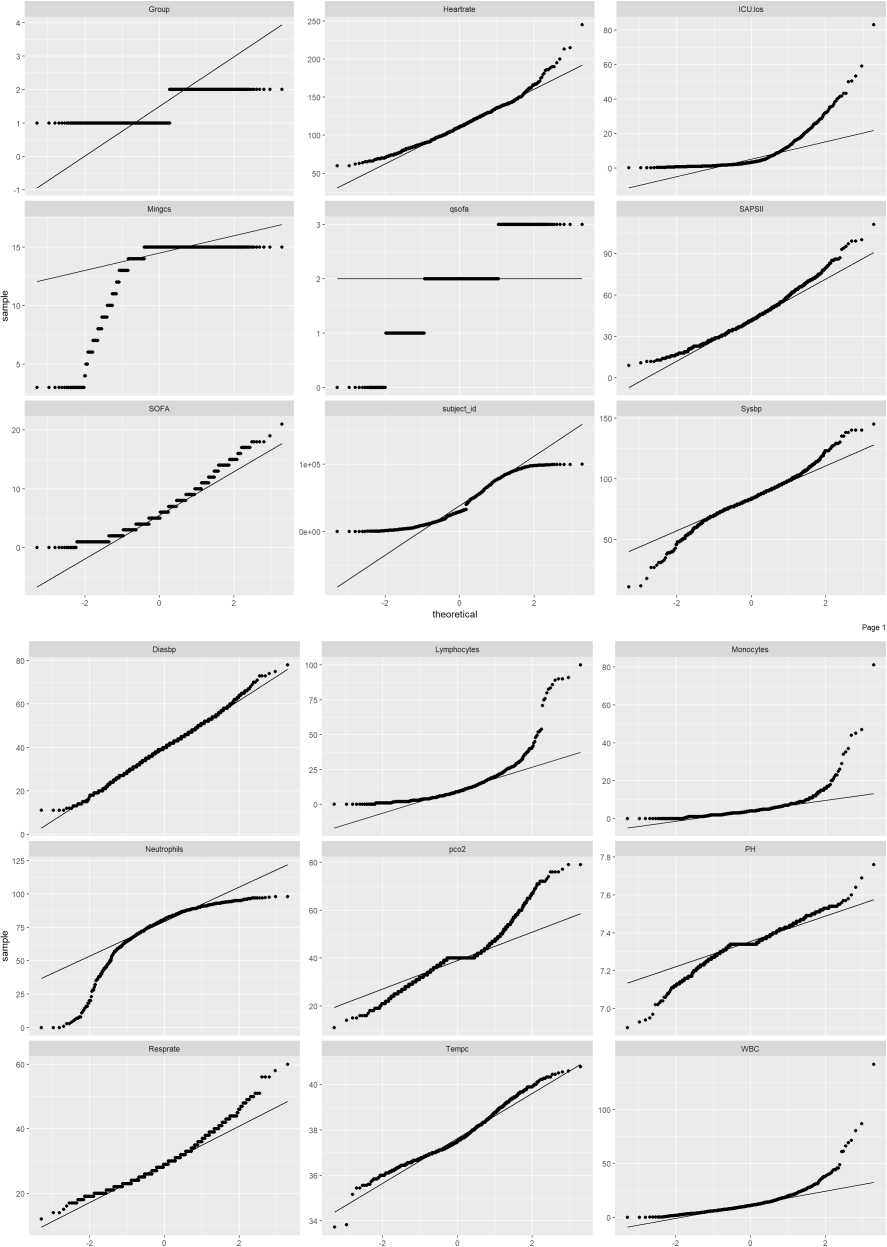


Page 2


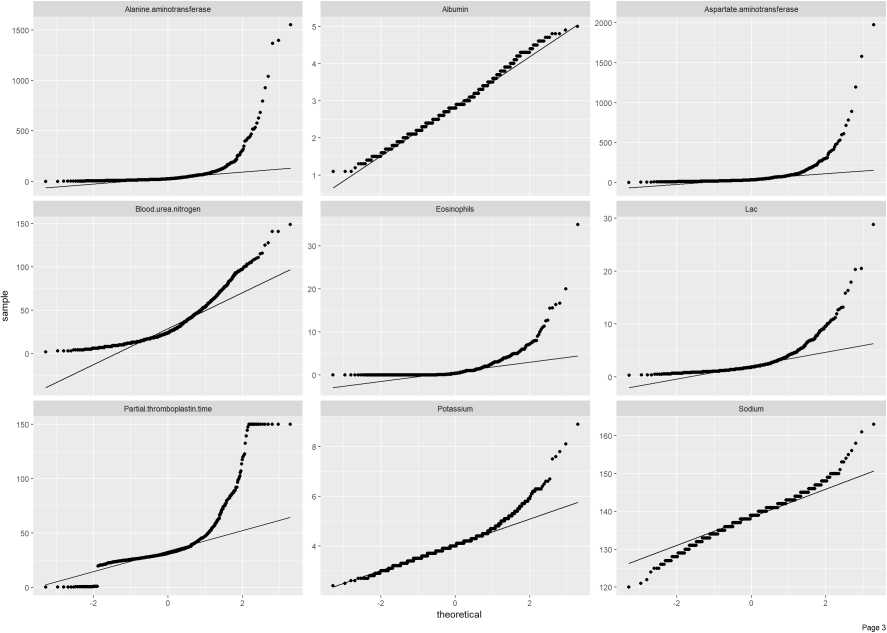


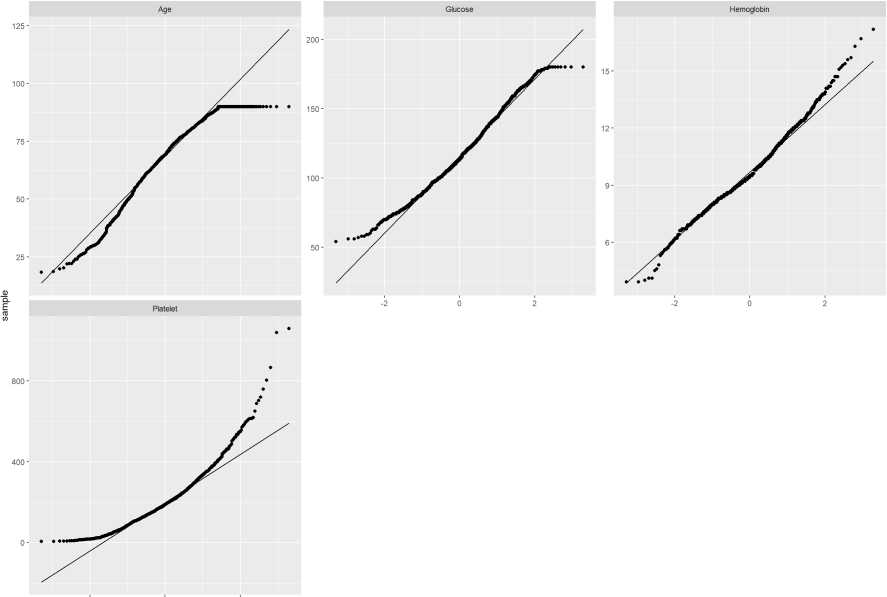


Correlation Analysis

Resprate

Tempe

subjected-

I I I I I I I I I I T I I I I I I I I subjectMiihgc£routCU.Ios|sofaSOFZ6APB^artraSfysb|E)ias^Espral<empcpco2 PH WBfibno摩e物岬白阈 Features

Gender_F

Blood.urea.nitrogen

Lymphocytes

Neutrophils


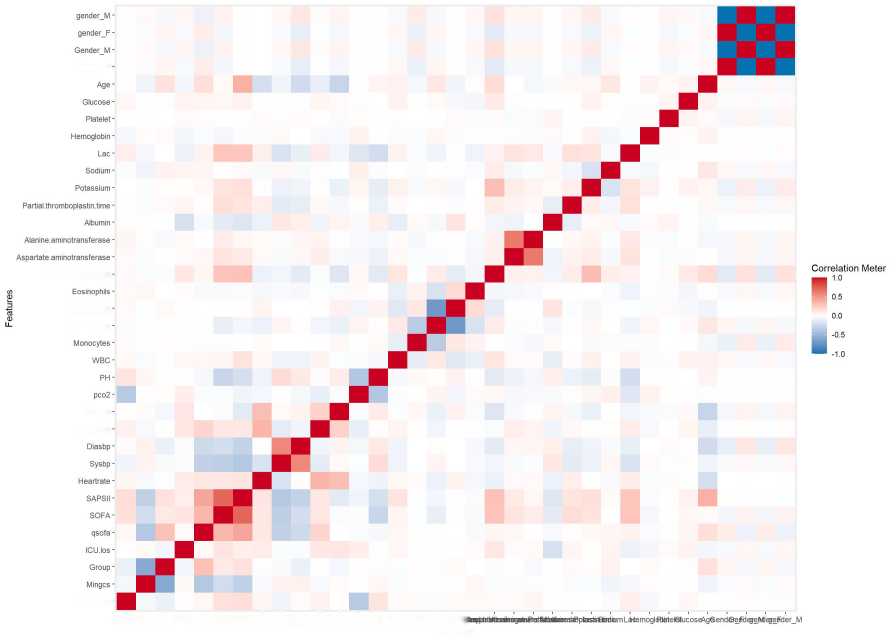


Principal Component Analysis

% Variance Explained By Principal Components

(Note: Labels indicate cumulative % explained variance)

2u(Duodluo。-Bd-OULId

12.0%

6.0%

% Variance Explained

Page 1


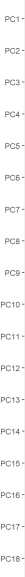

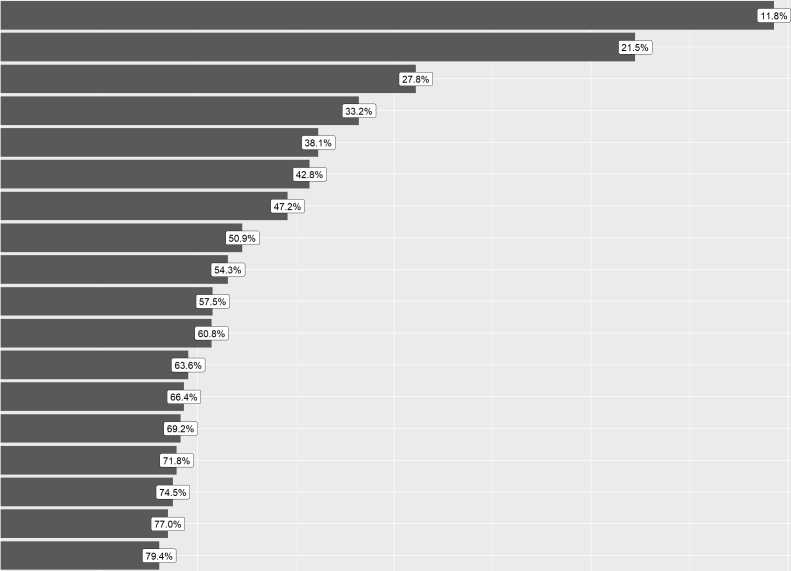

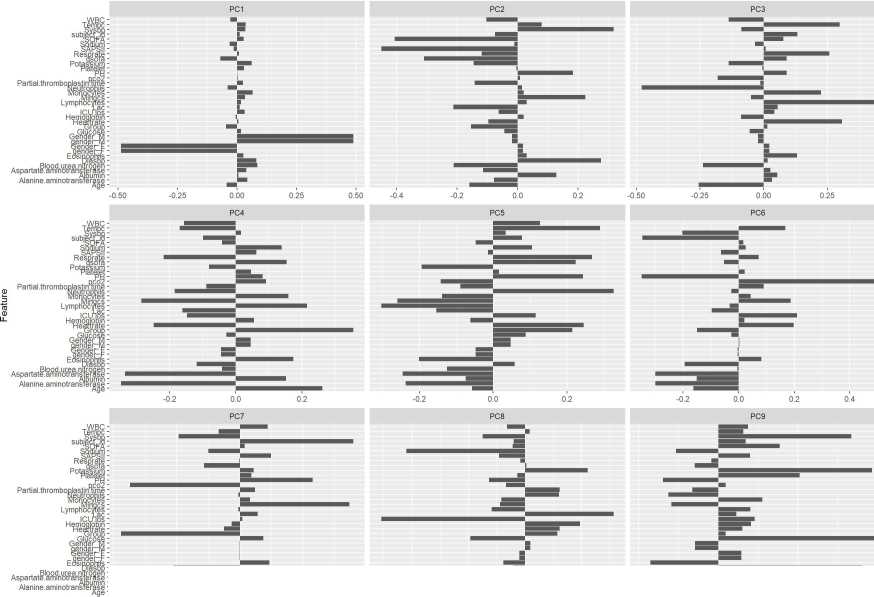

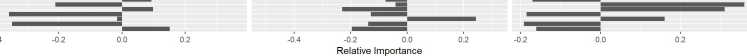


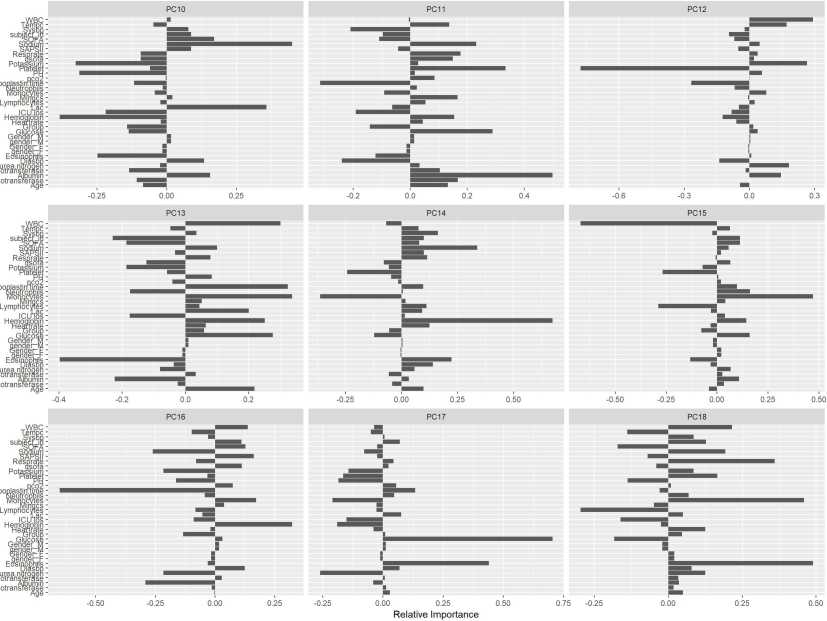


Page 2
