# Supplementary material for: Mechanical Learning for Prediction of Sepsis-Associated Encephalopathy
Source: Front Comput Neurosci. 2021 Nov 16;15:739265. doi: 10.3389/fncom.2021.739265 (PMC8636425; doi:10.3389/fncom.2021.739265)
Supplement: Supplementary Material 1 — Exclude patients with trauma of skull from the MIMIC-III database according to ICD9-codes. [file Data_Sheet_1.zip › Supplementary materials/Supplementary materials 4.DOCX]

| **Supplementary materials 4** Exclude patients with Epilepsy disease from the MIMIC III database according to ICD9-codes | | |
| --- | --- | --- |
| ICD9-code |  | Description |
| 34591 |  | Epilepsy, unspecified, with intractable epilepsy |
| 34570 |  | Epilepsia partialis continua, without mention of intractable epilepsy |
| 34571 |  | Epilepsia partialis continua, with intractable epilepsy |
| 34580 |  | Other forms of epilepsy and recurrent seizures, without mention of intractable epilepsy |
| 34581 |  | Other forms of epilepsy and recurrent seizures, with intractable epilepsy |
| 34590 |  | Epilepsy, unspecified, without mention of intractable epilepsy |
| 34591 |  | Epilepsy, unspecified, with intractable epilepsy |
| 64940 |  | Epilepsy complicating pregnancy, childbirth, or the puerperium, unspecified as to episode of care or not applicable |
| 64941 |  | Epilepsy complicating pregnancy, childbirth, or the puerperium, delivered, with or without mention of antepartum condition |
| 64942 |  | Epilepsy complicating pregnancy, childbirth, or the puerperium, delivered, with mention of postpartum complication |
| 64943 |  | Epilepsy complicating pregnancy, childbirth, or the puerperium, antepartum condition or complication |
| 64944 |  | Epilepsy complicating pregnancy, childbirth, or the puerperium, postpartum condition or complication |
