# Supplementary material for: Mechanical Learning for Prediction of Sepsis-Associated Encephalopathy
Source: Front Comput Neurosci. 2021 Nov 16;15:739265. doi: 10.3389/fncom.2021.739265 (PMC8636425; doi:10.3389/fncom.2021.739265)
Supplement: Supplementary Material 1 — Exclude patients with trauma of skull from the MIMIC-III database according to ICD9-codes. [file Data_Sheet_1.zip › Supplementary materials/Supplementary materials 5.DOCX]

| **Supplementary materials 5** Exclude patients with other cerebrovascular disease from the MIMIC III database according to ICD9-codes | | |
| --- | --- | --- |
| ICD9-code |  | Description |
| 3312 |  | Senile degeneration of brain |
| 3313 |  | Communicating hydrocephalus |
| 3314 |  | Obstructive hydrocephalus |
| 33189 |  | Other cerebral degeneration |
| 3319 |  | Cerebral degeneration, unspecified |
| 4378 |  | Other ill-defined cerebrovascular disease |
| 4379 |  | Unspecified cerebrovascular disease |
| 4380 |  | Unspecified cerebrovascular disease |
| 43810 |  | Late effects of cerebrovascular disease, speech and language deficit, unspecified |
