# Supplementary material for: Mechanical Learning for Prediction of Sepsis-Associated Encephalopathy
Source: Front Comput Neurosci. 2021 Nov 16;15:739265. doi: 10.3389/fncom.2021.739265 (PMC8636425; doi:10.3389/fncom.2021.739265)
Supplement: Supplementary Material 1 — Exclude patients with trauma of skull from the MIMIC-III database according to ICD9-codes. [file Data_Sheet_1.zip › Supplementary materials/Supplementary materials 6.DOCX]

| **Supplementary materials 6** Exclude patients with mental disorders and and neurological disease from the MIMIC III database according to ICD9-codes | | | | |  |
| --- | --- | --- | --- | --- | --- |
| ICD9-code |  | Description |  |  | |
| 29634 |  | Major depressive affective disorder, recurrent episode, severe, specified as with psychotic behavior |  |  | |
| 29635 |  | Major depressive affective disorder, recurrent episode, in partial or unspecified remission |  |  | |
| 29636 |  | Major depressive affective disorder, recurrent episode, in full remission |  |  | |
| 29640 |  | Bipolar I disorder, most recent episode (or current) manic, unspecified |  |  | |
| 29641 |  | Bipolar I disorder, most recent episode (or current) manic, mild |  |  | |
| 29642 |  | Bipolar I disorder, most recent episode (or current) manic, moderate |  |  | |
| 29643 |  | Bipolar I disorder, most recent episode (or current) manic, severe, without mention of psychotic behavior |  |  | |
| 29644 |  | Bipolar I disorder, most recent episode (or current) manic, severe, specified as with psychotic behavior |  |  | |
| 29645 |  | Bipolar I disorder, most recent episode (or current) manic, in partial or unspecified remission |  |  | |
| 29646 |  | Bipolar I disorder, most recent episode (or current) manic, in full remission |  |  | |
| 29650 |  | Bipolar I disorder, most recent episode (or current) depressed, unspecified |  |  | |
| 29651 |  | Bipolar I disorder, most recent episode (or current) depressed, mild |  |  | |
| 29652 |  | Bipolar I disorder, most recent episode (or current) depressed, moderate |  |  | |
| 29653 |  | Bipolar I disorder, most recent episode (or current) depressed, severe, without mention of psychotic behavior |  |  | |
| 29654 |  | Bipolar I disorder, most recent episode (or current) depressed, severe, specified as with psychotic behavior |  |  | |
| 29655 |  | Bipolar I disorder, most recent episode (or current) depressed, in partial or unspecified remission |  |  | |
| 29656 |  | Bipolar I disorder, most recent episode (or current) depressed, in full remission |  |  | |
| 29660 |  | Bipolar I disorder, most recent episode (or current) mixed, unspecified |  |  | |
| 29661 |  | Bipolar I disorder, most recent episode (or current) mixed, mild |  |  | |
| 29662 |  | Bipolar I disorder, most recent episode (or current) mixed, moderate |  |  | |
| 29663 |  | Bipolar I disorder, most recent episode (or current) mixed, severe, without mention of psychotic behavior |  |  | |
| 29664 |  | Bipolar I disorder, most recent episode (or current) mixed, severe, specified as with psychotic behavior |  |  | |
| 29665 |  | Bipolar I disorder, most recent episode (or current) mixed, in partial or unspecified remission |  |  | |
| 29666 |  | Bipolar I disorder, most recent episode (or current) mixed, in full remission |  |  | |
| 2967 |  | Bipolar I disorder, most recent episode (or current) unspecified |  |  | |
| 29680 |  | Bipolar disorder, unspecified |  |  | |
| 29681 |  | Atypical manic disorder |  |  | |
| 29682 |  | Atypical depressive disorder |  |  | |
| 29689 |  | Other bipolar disorders |  |  | |
| 30289 |  | Other specified psychosexual disorders |  |  | |
| 3029 |  | Unspecified psychosexual disorder |  |  | |
| 29682 |  | Atypical depressive disorder |  |  | |
| 2971 |  | Delusional disorder |  |  | |
| 2972 |  | Paraphrenia |  |  | |
| 2980 |  | Depressive type psychosis |  |  | |
| 2981 |  | Excitative type psychosis |  |  | |
| 2982 |  | Reactive confusion |  |  | |
| 2983 |  | Acute paranoid reaction |  |  | |
| 2984 |  | Psychogenic paranoid psychosis |  |  | |
| 2988 |  | Other and unspecified reactive psychosis |  |  | |
| 2989 |  | Unspecified psychosis |  |  | |
| 30111 |  | Chronic hypomanic personality disorder |  |  | |
| 30020 |  | Phobia, unspecified |  |  | |
| 30021 |  | Agoraphobia with panic disorder |  |  | |
| 30022 |  | Agoraphobia without mention of panic attacks |  |  | |
| 30023 |  | Social phobia |  |  | |
| 30029 |  | Other isolated or specific phobias |  |  | |
| 30112 |  | Chronic depressive personality disorder |  |  | |
| 30113 |  | Cyclothymic disorder |  |  | |
| 30120 |  | Schizoid personality disorder, unspecified |  |  | |
| 29381 |  | Psychotic disorder with delusions in conditions classified elsewhere |  |  | |
| 29410 |  | Dementia in conditions classified elsewhere without behavioral disturbance |  |  | |
| 29411 |  | Dementia in conditions classified elsewhere with behavioral disturbance |  |  | |
| 29420 |  | Dementia, unspecified, without behavioral disturbance |  |  | |
| 29421 |  | Dementia, unspecified, with behavioral disturbance |  |  | |
| 2949 |  | Unspecified persistent mental disorders due to conditions classified elsewhere |  |  | |
| 29500 |  | Simple type schizophrenia, unspecified |  |  | |
| 29501 |  | Simple type schizophrenia, subchronic |  |  | |
| 29502 |  | Simple type schizophrenia, chronic |  |  | |
| 29503 |  | Simple type schizophrenia, subchronic with acute exacerbation |  |  | |
| 29504 |  | Simple type schizophrenia, chronic with acute exacerbation |  |  | |
| 29505 |  | Simple type schizophrenia, in remission |  |  | |
| 29510 |  | Disorganized type schizophrenia, unspecified |  |  | |
| 29511 |  | Disorganized type schizophrenia, subchronic |  |  | |
| 29512 |  | Disorganized type schizophrenia, chronic |  |  | |
| 29513 |  | Disorganized type schizophrenia, subchronic with acute exacerbation |  |  | |
| 29514 |  | Disorganized type schizophrenia, chronic with acute exacerbation |  |  | |
| 29515 |  | Disorganized type schizophrenia, in remission |  |  | |
| 29520 |  | Catatonic type schizophrenia, unspecified |  |  | |
| 29521 |  | Catatonic type schizophrenia, subchronic |  |  | |
| 29522 |  | Catatonic type schizophrenia, chronic |  |  | |
| 29523 |  | Catatonic type schizophrenia, subchronic with acute exacerbation |  |  | |
| 29524 |  | Catatonic type schizophrenia, chronic with acute exacerbation |  |  | |
| 29525 |  | Catatonic type schizophrenia, in remission |  |  | |
| 29530 |  | Paranoid type schizophrenia, unspecified |  |  | |
| 29531 |  | Paranoid type schizophrenia, subchronic |  |  | |
| 29532 |  | Paranoid type schizophrenia, chronic |  |  | |
| 29533 |  | Paranoid type schizophrenia, subchronic with acute exacerbation |  |  | |
| 29534 |  | Paranoid type schizophrenia, chronic with acute exacerbation |  |  | |
| 29535 |  | Paranoid type schizophrenia, in remission |  |  | |
| 29540 |  | Schizophreniform disorder, unspecified |  |  | |
| 29541 |  | Schizophreniform disorder, subchronic |  |  | |
| 29542 |  | Schizophreniform disorder, chronic |  |  | |
| 29543 |  | Schizophreniform disorder, subchronic with acute exacerbation |  |  | |
| 29544 |  | Schizophreniform disorder, chronic with acute exacerbation |  |  | |
| 29545 |  | Schizophreniform disorder, in remission |  |  | |
| 29550 |  | Latent schizophrenia, unspecified |  |  | |
| 29551 |  | Latent schizophrenia, unspecified |  |  | |
| 29552 |  | Latent schizophrenia, chronic |  |  | |
| 29553 |  | Latent schizophrenia, subchronic with acute exacerbation |  |  | |
| 29554 |  | Latent schizophrenia, chronic with acute exacerbation |  |  | |
| 29555 |  | Latent schizophrenia, in remission |  |  | |
| 29560 |  | Schizophrenic disorders, residual type, unspecified |  |  | |
| 29561 |  | Schizophrenic disorders, residual type, subchronic |  |  | |
| 29562 |  | Schizophrenic disorders, residual type, chronic |  |  | |
| 29563 |  | Schizophrenic disorders, residual type, subchronic with acute exacerbation |  |  | |
| 29564 |  | Schizophrenic disorders, residual type, chronic with acute exacerbation |  |  | |
| 29565 |  | Schizophrenic disorders, residual type, in remission |  |  | |
| 29570 |  | Schizoaffective disorder, unspecified |  |  | |
| 29571 |  | Schizoaffective disorder, subchronic |  |  | |
| 29572 |  | Schizoaffective disorder, chronic |  |  | |
| 29573 |  | Schizoaffective disorder, subchronic with acute exacerbation |  |  | |
| 29574 |  | Schizoaffective disorder, chronic with acute exacerbation |  |  | |
| 29580 |  | Other specified types of schizophrenia, unspecified |  |  | |
| 29581 |  | Other specified types of schizophrenia, subchronic |  |  | |
| 29582 |  | Other specified types of schizophrenia, chronic |  |  | |
| 29583 |  | Other specified types of schizophrenia, subchronic with acute exacerbation |  |  | |
| 29584 |  | Other specified types of schizophrenia, chronic with acute exacerbation |  |  | |
| 29585 |  | Other specified types of schizophrenia, in remission |  |  | |
| 29590 |  | Unspecified schizophrenia, unspecified |  |  | |
| 29591 |  | Unspecified schizophrenia, subchronic |  |  | |
| 29592 |  | Unspecified schizophrenia, chronic |  |  | |
| 29593 |  | Unspecified schizophrenia, subchronic with acute exacerbation |  |  | |
| 29594 |  | Unspecified schizophrenia, chronic with acute exacerbation |  |  | |
| 29595 |  | Unspecified schizophrenia, in remission |  |  | |
| 29600 |  | Bipolar I disorder, single manic episode, unspecified |  |  | |
| 29601 |  | Bipolar I disorder, single manic episode, mild |  |  | |
| 29602 |  | Bipolar I disorder, single manic episode, moderate |  |  | |
| 29603 |  | Bipolar I disorder, single manic episode, severe, without mention of psychotic behavior |  |  | |
| 29604 |  | Bipolar I disorder, single manic episode, severe, specified as with psychotic behavior |  |  | |
| 29605 |  | Bipolar I disorder, single manic episode, in partial or unspecified remission |  |  | |
| 29606 |  | Bipolar I disorder, single manic episode, in full remission |  |  | |
| 29610 |  | Manic affective disorder, recurrent episode, unspecified |  |  | |
| 29611 |  | Manic affective disorder, recurrent episode, mild |  |  | |
| 29612 |  | Manic affective disorder, recurrent episode, moderate |  |  | |
| 29613 |  | Manic affective disorder, recurrent episode, severe, without mention of psychotic behavior |  |  | |
| 29614 |  | Manic affective disorder, recurrent episode, severe, specified as with psychotic behavior |  |  | |
| 29615 |  | Manic affective disorder, recurrent episode, in partial or unspecified remission |  |  | |
| 29616 |  | Manic affective disorder, recurrent episode, in full remission |  |  | |
| 29620 |  | Major depressive affective disorder, single episode, unspecified |  |  | |
| 29621 |  | Major depressive affective disorder, single episode, mild |  |  | |
| 29622 |  | Major depressive affective disorder, single episode, moderate |  |  | |
| 29623 |  | Major depressive affective disorder, single episode, severe, without mention of psychotic behavior |  |  | |
| 29624 |  | Major depressive affective disorder, single episode, severe, specified as with psychotic behavior |  |  | |
| 29625 |  | Major depressive affective disorder, single episode, in partial or unspecified remission |  |  | |
| 29626 |  | Major depressive affective disorder, single episode, in full remission |  |  | |
| 29630 |  | Major depressive affective disorder, recurrent episode, unspecified |  |  | |
| 29631 |  | Major depressive affective disorder, recurrent episode, mild |  |  | |
| 29632 |  | Major depressive affective disorder, recurrent episode, moderate |  |  | |
| 29633 |  | Major depressive affective disorder, recurrent episode, severe, without mention of psychotic behavior |  |  | |
| 33182 |  | Dementia with lewy bodies |  |  | |
| 3310 |  | Alzheimer's disease |  |  | |
| 33119 |  | Other frontotemporal dementia |  |  | |
| 64842 |  | Mental disorders of mother, delivered, with mention of postpartum complication |  |  | |
| 64843 |  | Mental disorders of mother, antepartum condition or complication |  |  | |
| 64844 |  | Mental disorders of mother, postpartum condition or complication |  |  | |
| 3181 |  | Severe intellectual disabilities |  |  | |
| 3182 |  | Profound intellectual disabilities |  |  | |
| 33182 |  | Dementia with lewy bodies |  |  | |
