# Supplementary material for: Mechanical Learning for Prediction of Sepsis-Associated Encephalopathy
Source: Front Comput Neurosci. 2021 Nov 16;15:739265. doi: 10.3389/fncom.2021.739265 (PMC8636425; doi:10.3389/fncom.2021.739265)
Supplement: Supplementary Material 1 — Exclude patients with trauma of skull from the MIMIC-III database according to ICD9-codes. [file Data_Sheet_1.zip › Supplementary materials/Supplementary materials 7.DOCX]

| **Supplementary materials 7** Exclude patients with alcoholic intoxication or or drug abuse from the MIMIC III database according to ICD9-codes | | |
| --- | --- | --- |
| ICD9-code |  | Description |
| 30303 |  | Acute alcoholic intoxication in alcoholism, in remission |
| 30390 |  | Other and unspecified alcohol dependence, unspecified |
| 30391 |  | Other and unspecified alcohol dependence, continuous |
| 30392 |  | Other and unspecified alcohol dependence, episodic |
| 30393 |  | Other and unspecified alcohol dependence, in remission |
| 30400 |  | Opioid type dependence, unspecified |
| 30401 |  | Opioid type dependence, continuous |
| 30402 |  | Opioid type dependence, episodic |
| 30403 |  | Opioid type dependence, in remission |
| 30410 |  | Sedative, hypnotic or anxiolytic dependence, unspecified |
| 30411 |  | Sedative, hypnotic or anxiolytic dependence, continuous |
| 30412 |  | Sedative, hypnotic or anxiolytic dependence, episodic |
| 30413 |  | Sedative, hypnotic or anxiolytic dependence, in remission |
| 30420 |  | Cocaine dependence, unspecified |
| 30421 |  | Cocaine dependence, continuous |
| 30422 |  | Cocaine dependence, episodic |
| 30423 |  | Cocaine dependence, in remission |
| 30430 |  | Cannabis dependence, unspecified |
| 30431 |  | Cannabis dependence, continuous |
| 30432 |  | Cannabis dependence, episodic |
| 30433 |  | Cannabis dependence, in remission |
| 30440 |  | Amphetamine and other psychostimulant dependence, unspecified |
| 30441 |  | Amphetamine and other psychostimulant dependence, continuous |
| 30442 |  | Amphetamine and other psychostimulant dependence, episodic |
| 30443 |  | Amphetamine and other psychostimulant dependence, in remission |
| 30450 |  | Hallucinogen dependence, unspecified |
| 30451 |  | Hallucinogen dependence, continuous |
| 30452 |  | Hallucinogen dependence, episodic |
| 30453 |  | Hallucinogen dependence, in remission |
| 30460 |  | Other specified drug dependence, unspecified |
