# Supplementary material for: Mechanical Learning for Prediction of Sepsis-Associated Encephalopathy
Source: Front Comput Neurosci. 2021 Nov 16;15:739265. doi: 10.3389/fncom.2021.739265 (PMC8636425; doi:10.3389/fncom.2021.739265)
Supplement: Supplementary Material 1 — Exclude patients with trauma of skull from the MIMIC-III database according to ICD9-codes. [file Data_Sheet_1.zip › Supplementary materials/Supplementary materials 8 .DOCX]

| **Supplementary materials 8** Exclude patients with metabolic encephalopathy, hepatic encephalopathy,hypertensive encephalopathy,diabetes with coma, disorders of urea cycle, hypernatremia from the MIMIC III database according to ICD9-codes | | |
| --- | --- | --- |
| ICD9-codes |  | Description |
| 34831 |  | Metabolic encephalopathy |
| 5722 |  | Hepatic encephalopathy |
| 700 |  | Viral hepatitis A with hepatic coma |
| 7020 |  | Viral hepatitis B with hepatic coma, acute or unspecified, without mention of hepatitis delta |
| 7021 |  | Viral hepatitis B with hepatic coma, acute or unspecified, with hepatitis delta |
| 7022 |  | Chronic viral hepatitis B with hepatic coma without hepatitis delta |
| 7023 |  | Chronic viral hepatitis B with hepatic coma with hepatitis delta |
| 7041 |  | Acute hepatitis C with hepatic coma |
| 7042 |  | Hepatitis delta without mention of active hepatitis B disease with hepatic coma |
| 7043 |  | Hepatitis E with hepatic coma |
| 7044 |  | Chronic hepatitis C with hepatic coma |
| 7049 |  | Other specified viral hepatitis with hepatic coma |
| 7052 |  | Hepatitis delta without mention of active hepatitis B disease or hepatic coma |
| 706 |  | Unspecified viral hepatitis with hepatic coma |
| 7071 |  | Unspecified viral hepatitis C with hepatic coma |
| 2706 |  | Disorders of urea cycle metabolism |
| 2510 |  | Hypoglycemic coma |
| 4372 |  | Hypertensive encephalopathy |
